# Omics analysis of *Mycobacterium tuberculosis* isolates uncovers Rv3094c, an ethionamide metabolism-associated gene

**DOI:** 10.1038/s42003-023-04433-w

**Published:** 2023-02-07

**Authors:** Li Wan, Peilei Hu, Lili Zhang, Zhao-Xi Wang, Joy Fleming, Bo Ni, Jianjun Luo, Cha-Xiang Guan, Liqiong Bai, Yunhong Tan, Haican Liu, Na Li, Tongyang Xiao, Hua Bai, Yong-An Zhang, Xian-En Zhang, Kanglin Wan, Lijun Bi, Songying Ouyang, Hongtai Zhang

**Affiliations:** 1 Key Laboratory of RNA Biology, Institute of Biophysics, Chinese Academy of Sciences, Beijing, 100101 China; 2 Provincial University Key Laboratory of Cellular Stress Response and Metabolic Regulation, the Key Laboratory of Innate Immune Biology of Fujian Province, Biomedical Research Center of South China, Key Laboratory of OptoElectronic Science and Technology for Medicine of the Ministry of Education, Fujian Key Laboratory of Special Marine Bio-resources Sustainable Utilization, College of Life Sciences, Fujian Normal University, Fuzhou, 350117 Fujian Province China; 3 State Key Laboratory for Infectious Diseases Prevention and Control, Collaborative Innovation Center for Diagnosis and Treatment of Infectious Diseases, National Institute for Communicable Disease Control and Prevention, Chinese Center for Disease Control and Prevention, Beijing, 102206 China; 4 Department of Physiology, Xiangya School of Medicine, Central South University, Changsha, Hunan 410078 China; 5 Hunan Chest Hospital, Changsha, 410013 Hunan Province China; 6 University of Chinese Academy of Sciences, Beijing, 100049 China; 7 National Laboratory of Biomacromolecules, Institute of Biophysics, Chinese Academy of Sciences, Beijing, 100101 China; 8 Guangdong Province Key Laboratory of TB Systems Biology and Translational Medicine, Foshan, 528000 Guangdong Province China; 9 Beijing Center for Disease Prevention and Control, Beijing, 100013 China

**Keywords:** Antimicrobial resistance, Clinical microbiology, Pathogens

## Abstract

Global control of the tuberculosis epidemic is threatened by increasing prevalence of drug resistant *M. tuberculosis* isolates. Many genome-wide studies focus on SNP-associated drug resistance mechanisms, but drug resistance in 5–30% of *M. tuberculosis* isolates (varying with antibiotic) appears unrelated to reported SNPs, and alternative drug resistance mechanisms involving variation in gene/protein expression are not well-studied. Here, using an omics approach, we identify 388 genes with lineage-related differential expression and 68 candidate drug resistance-associated gene pairs/clusters in 11 *M. tuberculosis* isolates (variable lineage/drug resistance profiles). Structural, mutagenesis, biochemical and bioinformatic studies on Rv3094c from the Rv3093c-Rv3095 gene cluster, a gene cluster selected for further investigation as it contains a putative monooxygenase/repressor pair and is associated with ethionamide resistance, provide insights on its involvement in ethionamide sulfoxidation, the initial step in its activation. Analysis of the structure of Rv3094c and its complex with ethionamide and flavin mononucleotide, to the best of our knowledge the first structures of an enzyme involved in ethionamide activation, identify key residues in the flavin mononucleotide and ethionamide binding pockets of Rv3094c, and F221, a gate between flavin mononucleotide and ethionamide allowing their interaction to complete the sulfoxidation reaction. Our work broadens understanding of both lineage- and drug resistance-associated gene/protein expression perturbations and identifies another player in mycobacterial ethionamide metabolism.

## Abstract (web summary)

A multi-omic analysis of 11 *Mycobacterium tuberculosis* (Mtb) isolates with different drug resistance profiles leads to the discovery of Rv3094c, an enzyme capable of activating the clinically relevant Mtb drug, ethionamide.

## Introduction

Prior to the COVID-19 pandemic, tuberculosis, caused by the ancient pathogen *Mycobacterium tuberculosis*, was the leading cause of death by infectious diseases, with ~10 million new cases and 1.5 million deaths occurring worldwide in 2020^1^. Drug-resistant tuberculosis is a major public health threat; 214,000 drug-resistant tuberculosis-related deaths occurred in 2018, and ~78% of the world’s 484,000 drug-resistant tuberculosis cases had multi-drug-resistant tuberculosis, ~6.2% of these having extensively-drug resistant tuberculosis^2,3^. Global treatment success rates vary from 85% for drug-sensitive tuberculosis, to 56% for multi-drug resistant tuberculosis, to 39% for extensively-drug resistant tuberculosis^2,3^. A more comprehensive understanding of drug resistance mechanisms is key for designing drugs to circumvent existing resistance mechanisms or exploit alternative druggable biochemical pathways.

Whole genome sequencing approaches are increasingly used to identify and investigate drug resistance mechanisms, focusing largely on how single nucleotide polymorphisms (SNPs) in drug target genes lead to drug resistance^4^. Such SNPs have predictive value and are exploited in the development of molecular drug sensitivity tests for clinical use (reviewed elsewhere^5^). However, drug resistance mechanisms in 5–30% drug-resistant isolates (rifampin: <5%; isoniazid: 5%; pyrazinamide and amikacin: 10%; ethionamide: 15%; ethambutol: 30%) are unknown^6^, and the downstream ripple effects of SNPs on the transcriptome, proteome and general metabolism of the bacterial cell are rarely considered. Non-SNP-associated drug resistance mechanisms are known^7^ and include production of enzymes such as β-lactamases that modify or inactivate drugs^8^, pumping out of drugs through efflux systems^9^, alterations of cell permeability^10^, and dysfunction of genome stability systems^11^. Evidence for drug resistance mechanisms involving changes in gene transcription or protein expression levels has also been reported; for example, overexpression of *inhA* confers resistance to isoniazid and ethionamide in *M. smegmatis*, *M. bovis* BCG, and *M. tuberculosis*^12^, and overexpression of Rv2459 (*jefA*) in *M. tuberculosis* H37Ra leads to an increase in the minimum inhibitory concentration of first-line drugs such as isoniazid and ethionamide^13^.

Genetic diversity in *M. tuberculosis* is low due to its clonal nature^14^. The *M. tuberculosis* complex (MTBC) is classified into seven phylogenetic lineages (L1-7) associated with different geographical regions based on an SNP barcoding system^15^. Lineage L2 (East Asian) and L4 (Euro-American) isolates have the widest distribution and show phenotypic differences that impact their capacity to cause disease and develop drug resistance^16,17^. Beijing genotypes (L2), for example, are associated with a higher prevalence of drug resistance and higher rates of disease transmission^16^. Reports indicating that the evolutionary trajectories of different lineages may be diverging in response to antibiotic therapy^18^ indicate the importance of considering the impact of lineage on analyses designed to detect previously unknown drug resistance mechanisms.

Here, to gain insight into drug resistance mechanisms not directly related to drug target gene SNPs, and identify potentially druggable pathways, we used an omics approach to analyze transcriptomic and proteomic variation in 11 isolates from two lineages (L2 and L4) varying in drug resistance profiles. We find that perturbations in gene/protein expression likely contribute to the development of drug resistance, and also make an important contribution to phenotypic differences between *M. tuberculosis* lineages. Our omics analysis points to the involvement of 68 gene pairs/clusters in drug resistance in this set of isolates. Our subsequent investigation of one gene cluster (Rv3093c-Rv3095) associated with ethionamide resistance that contained a putative monooxygenase/repressor pair with some resemblance to *ethA/ethR*, indicates that Rv3094c is likely a flavin-dependent monooxygenase that sulfoxygenates ethionamide, and Rv3095 is its transcriptional regulator. Using structural studies of Rv3094c and its complex with ethionamide and flavin mononucleotide, we identify key residues in its ethionamide and flavin mononucleotide binding pockets and provide insight into its ethionamide sulfoxidation mechanism. Our work broadens understanding of the role gene/protein expression perturbations play in drug resistance mechanisms and may open up another avenue of research on ethionamide metabolism in mycobacteria.

## Results

### Omics approach

**Fig. 1:**
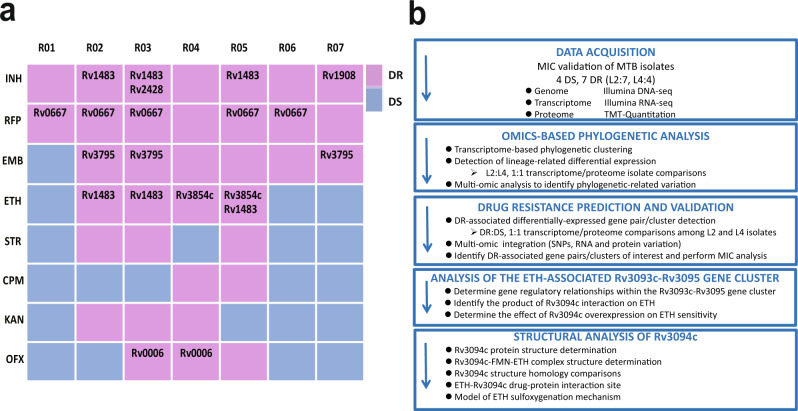
Study outline and workflow for the analysis of 11 *M. tuberculosis* isolates with different drug resistance profiles. **a** Drug resistance profiles and SNP-containing well-established drug resistance-associated genes present in the seven drug-resistant isolates. Gene IDs are those of well-established drug resistance-associated genes containing SNPs present in the isolate. **b** Study outline and workflow (drug-sensitive: DS; drug-resistant: DR).

Conventional approaches in genomics-based drug resistance studies have focused on finding high-frequency mutations in drug-resistant isolates. However, mechanisms of resistance in 5–30% of drug-resistant isolates (varying with antibiotic) are unknown^19^. As the complex drug resistance genetic background of clinical isolates can make it difficult to match specific drug resistances to specific well-established drug resistance-associated SNP-containing genes, we reasoned that considering the role variation in gene/protein expression plays in drug resistance mechanisms may uncover as yet unknown paths to drug resistance. To increase the likelihood of uncovering genes/proteins associated with drug resistance mechanisms involving altered gene/protein expression rather than mechanisms based on drug target gene mutations, we selected 11 previously *sequenced M. tuberculosis* isolates^20^ with different drug resistance profiles (drug-sensitive isolates S01 (H37Rv reference strain) and S02-04, multi-drug-resistant isolates R01, R06-07, and extensively drug-resistant isolates R02-05; Supplementary Data 1) that lacked many well-known drug resistance-associated SNPs and had unexplained resistance mechanisms. The isolates chosen had SNPs in 7 well-established SNP-containing resistance genes (*gyrA* Rv0006, *rpoB* Rv0667, *fabG1* Rv1483, *katG* Rv1908c, *ahpC* Rv2428, *embB* Rv3795, and *ethA* Rv3854c)^21^ (Supplementary Data 2; Fig. 1a), but their presence did not fully explain observed resistances. For example, isolates R04 and R07 show rifampin resistance, but have no resistance-associated mutations in the RRDR (rifampicin resistance determining region) of *rpoB* (Rv0667), and isoniazid-resistant isolates R01, R04, and R06 have no resistance-associated mutations in *katG* (Rv1908c), *ahpC* (Rv2428), *fabG1* (Rv1483), or *inhA* (Rv1484). We performed RNA-seq and TMT quantitative proteomic analysis on these isolates in the absence of drug stimulus to minimize the confounding influence of different resistances in the complex genetic background of clinical isolates, and to hone in on the native physiological and biochemical mechanisms/states that underlie drug resistance, and then compared their respective genomes, transcriptomes and proteomes (Fig. 1b).

A total of 1402 SNPs were detected, using *M. tuberculosis* H37Rv (NC_000962) as the reference genome (Supplementary Data 2). Transcripts from 3915 of the 4031 predicted *M. tuberculosis* genes were detected in all samples (Supplementary Data 3), and 3019 proteins were detected, 2099 being common to all samples (Supplementary Data 4).

### Phylogenetic relationships are reflected in the transcriptome and proteome

**Fig. 2:**
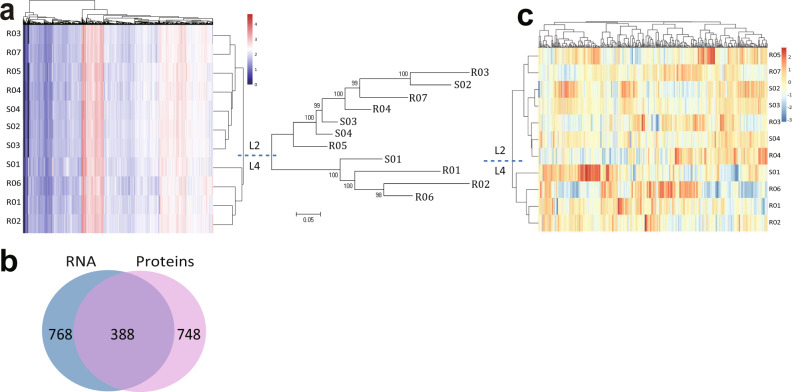
Phylogenetic relationships are reflected in the transcriptome and proteome. **a** Hierarchical clustering of transcriptome data from 11 *M. tuberculosis* isolates and phylogenetic analysis of the same isolates based on SNP variation. The scale bar represents the number of SNP substitutions per SNP site. **b** Venn diagram showing the number of genes that are differentially expressed at both the RNA (Log_2_ (fold change) >1 or −1, *p* < 0.05) and protein (Log_2_ (fold change) >1 or <−1, *p* < 0.05) levels in L2:L4 strain comparisons. **c** Hierarchical clustering of the 11 isolates using transcriptomic data from the 388 overlapping genes differentially-expressed at the transcriptome and proteome levels.

Before performing analyses to detect unknown drug resistance associations, we examined relationships between gene/protein expression and lineage. Hierarchical clustering of the 11 transcriptome profiles revealed a close correlation in gene expression levels among L2 isolates and among L4 isolates, reflecting phylogenetic relationships similarly to hierarchical clustering based on SNP variation (Fig. 2a), and suggesting that variation in gene expression levels in *M. tuberculosis* may be subject to the systematic influence of lineage-related SNPs and reflect phylogenetic relationships.

To identify genes whose expression is lineage-related, we compared the transcriptome and proteome of each L2 with each L4 isolate (Supplementary Data 5). 388 genes showed differential expression in both the transcriptome and proteome in at least one comparison (Log fold-change >1 or <−1, *p*-value <0.05) (Fig. 2b; Supplementary Data 6). Hierarchical clustering of the 11 transcriptomes using only these 388 genes clustered the isolates successfully into L2 and L4 lineages (Fig. 2c), suggesting the influence of lineage-related SNPs is largely reflected in the expression of the 388 genes. Genes selected by this multi-omics approach that vary at both omic levels may thus be closely linked to lineage-related phenotypic variation between isolates.

GO function analysis of the 172/388 genes for which data was available showed that enriched functions included metabolic processes and pathogenesis (*p* < 0.05). KEGG pathway analysis pointed to enrichment in ABC transporter, inositol phosphate metabolism, and ether lipid metabolism genes (*p* < 0.05) (Supplementary Fig. 1a; Supplementary Data 7).

Genomic variation between isolates (1402 SNPs) was widely dispersed throughout the genome; we identified 130 lineage-related SNPs scattered among 113 genes and 16 intergenic regions (Supplementary Data 2). However, only 9 of the above 388 lineage-related differential genes and 6 intergenic regions contained lineage-related SNPs (Supplementary Data 6). Hierarchical clustering based on gene expression data for the remaining 373 genes still accurately reflected phylogenetic relationships (Supplementary Fig. 1b), suggesting that genes containing lineage-related SNPs do not determine phylogenetic relationships directly. Transcriptomic variation thus appears to have a strong relationship with lineage, especially with genes whose expression varies in both the transcriptome and proteome. This lineage-related variation based on variation in gene/protein expression rather than in SNP-carrying genes may contribute to the differences in pathogenic characteristics between *M. tuberculosis* lineages.

### Selection of candidate drug resistance-associated gene pair/clusters

**Fig. 3:**
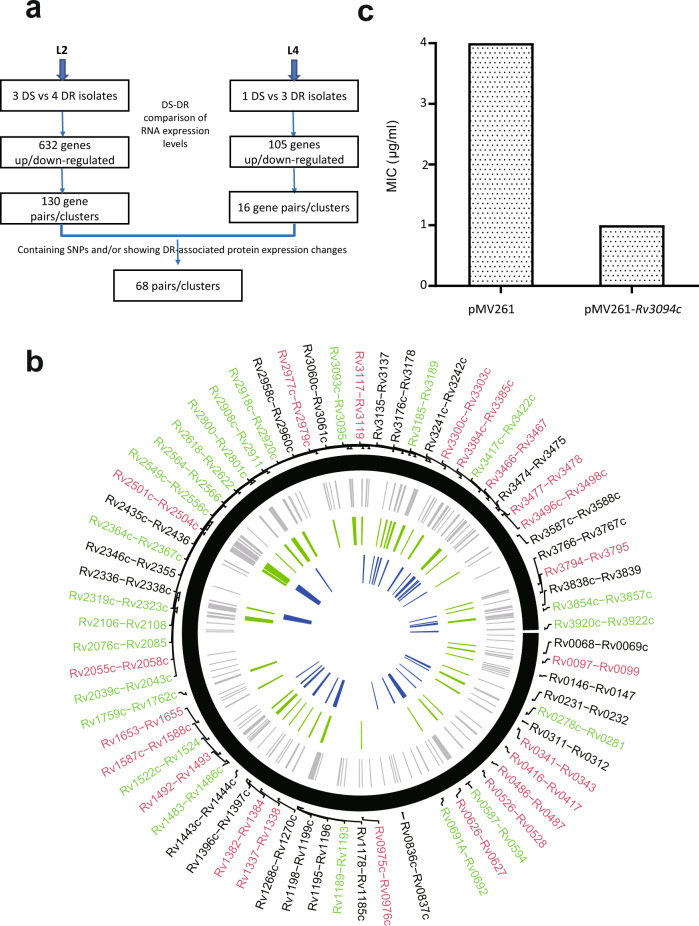
Multi-omic analysis identifies 68 candidate drug resistance-associated gene pairs/clusters. **a** Flowchart of the analysis of transcriptomic/genomic/proteomic data from the 11 isolates leading to the identification of drug resistance-associated gene pairs/clusters. **b** Distribution of candidate drug resistance-associated gene pairs/clusters showing multi-omic variation in the *M. tuberculosis* genome. Outermost circle: distribution of the 68 drug resistance-associated gene pairs/clusters in the *M. tuberculosis* genome (black: gene pair/cluster does not contain an operon; red: all genes in the pair/cluster are in the same operon; green: some genes in the pair/cluster are in the same operon), the second circle (gray) shows the 144 gene pairs/clusters that show drug resistance-associated variation in RNA levels. The third circle (green) shows 48 gene pairs/clusters that had drug resistance-associated changes in protein expression and the innermost circle (blue) shows 38 genes pairs/clusters that contain candidate drug resistance-associated SNPs. Results were visualized using Circus^63^. **c** MIC analysis of an *M. tuberculosis* H37Ra strain overexpressing Rv3094c against ethionamide. Results presented are representative of three replicate experiments.

Genomic variation among strains leads to variation in gene/protein expression levels that in turn lead to variation in phenotype. Just as SNPs occur randomly, variation in gene expression levels also has a random element and is influenced by many factors both related and unrelated to drug resistance^16^. In bacteria, genes associated with related functions/metabolic pathways are often located adjacently in pairs/clusters and transcribed as a single transcriptional unit (operon). For example, Rv0341-Rv0342 (*iniB-iniA*) associated with isoniazid and ethambutol tolerance^22^, and Rv3854-Rv3855 (*ethA-ethR*) associated with ethionamide resistance^23^, both of which have mechanisms involving changes in gene/protein expression, are located in adjacent pairs/clusters. To increase the probability of identifying unknown genes/proteins associated with drug resistance mechanisms involving perturbations in gene/protein expression, we thus searched for the subset of drug resistance-associated genes that were simultaneously differentially-expressed in pairs/clusters (Log foldchange >1 or <−1, *p*-value <0.05) in drug resistant: drug sensitive isolate transcriptome comparisons (Fig. 3a), minimizing the influence of lineage-related variation by restricting comparative analyses to L2 or L4 isolates, SNPs, gene expression and protein levels in each drug-resistant isolate being compared with each drug sensitive isolate in the same lineage. 144 gene pairs/clusters (L2: 130, L4: 16) showed simultaneous gene expression changes (upregulation or downregulation) in ≥1 drug resistant: drug-sensitive isolate comparison and were located adjacently as pairs/clusters in the genome (Supplementary Data 8). We reasoned that gene pairs/clusters most closely associated with drug resistance would contain genes that also (1) showed drug resistance-associated protein expression changes (upregulation or downregulation) and/or (2) contained SNPs in drug-resistant isolates (Fig. 3b; Supplementary Data 9). While these criteria excluded well-known resistance-associated SNP-containing drug target genes such as *rpoB*, *katG*, *gyrA*, and *gyrB*^21^, 68 gene pairs/clusters encompassing a total of 231 genes were selected, including gene pairs *iniB-iniA*, *inhA-hemZ*, *embA-embB*, and *ethA-ethR*, whose resistance mechanisms are related to changes in gene expression^23,24^.

GO/KEGG analysis of the 109/231 genes for which data was available indicated that these gene pairs/clusters were enriched in phospholipid catabolic processes, and response to antibiotics (*p* < 0.05) and ABC transporters, inositol phosphate metabolism, and ether lipid metabolism pathways (*p* < 0.05) (Supplementary Fig. 2; Supplementary Data 10). Interestingly, genes in 52/68 gene pairs/clusters were predicted (Operon-mapper^25^) to be co-transcribed from the same putative operon (Fig. 3b; Supplementary Data 11), and 7 gene pairs/clusters, including the *ethA*/*ethR* gene pair associated with ethionamide metabolism, included genes encoding regulatory proteins (Supplementary Data 9).

To evaluate the potential involvement of the gene pairs/clusters in drug resistance and identify pathways of interest for further investigation, we examined their gene annotations and performed minimal inhibitory concentration (MIC) analysis (Supplementary Data 12) on a selection of genes from 11/68 gene pairs/clusters for seven anti-tubercular drugs (rifampin, isoniazid, streptomycin, ethambutol, ofloxacin, capreomycin and ethionamide), including genes whose resistance mechanisms are known to involve altered gene expression (*iniA*, *iniB*, *inhA*, *hemZ*, *embA*, *embB*, *ethA*, *ethR*) as controls. As the presence of redundant pathways and complementary mechanisms would mask the effects of gene knockouts, we overexpressed the selected genes in the drug sensitive *M. tuberculosis* laboratory strain H37Ra using the pMV261 extrachromosomal low copy-number plasmid so that resistance associations could be evaluated one at a time outside the complex resistance genetic background of clinical isolates. Eighteen of the 27 genes tested (66.7%) had resistance associations with ≥1 drug (≥2 or ≤−2-fold increases/decreases in MIC values; Supplementary Data 12).

We noted that Rv3094c overexpression conferred hypersensitivity to ethionamide, inducing a 4-fold reduction in its MIC (Fig. 3c; Supplementary Data 13). Ethionamide is a second-line anti-tubercular drug used to treat patients infected with multi-drug-resistant or extensively drug-resistant isolates, and is a pro-drug that requires metabolic activation before it can exert its cytotoxic effects. EthA, a Type I Baeyer-Villiger monooxygenase (BVMO), is generally considered to be the main activator of ethionamide^26^, and is repressed by the binding of EthR to the intergenic region between *ethA* and *ethR*, ultimately repressing ethionamide activation and promoting cell survival^27^. The annotations of genes in the Rv3093c-Rv3095 cluster (Rv3093c: an FAD/FMN (flavin adenine dinucleotide/flavin mononucleotide) reductase; Rv3094c: a hypothetical protein predicted to belong to the FAD-containing Acyl-CoA dehydrogenase (ACAD) superfamily and participate in oxidation-reduction reactions; and Rv3095: a hypothetical HTH transcriptional regulator) suggested some similarity to the *ethA/ethR* gene pair, the presence of a putative monooxygenase/repressor pair leading us to wonder if the Rv3093c-Rv3095 cluster might have an as yet uninvestigated role in ethionamide metabolism. We thus focussed our attention on investigating the potential involvement of this gene cluster in ethionamide metabolism.

### Rv3094c sulfoxygenates ethionamide

**Fig. 4:**
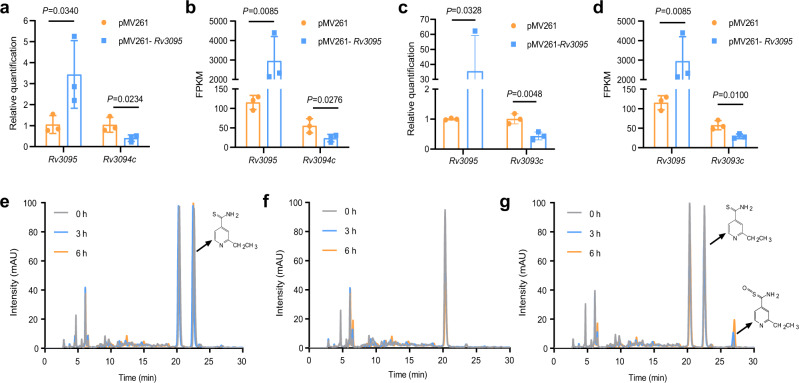
Gene pair Rv3094c-Rv3095 is associated with ethionamide metabolism. **a**–**d** Analysis of Rv3094c (**a**, **b**) and Rv3093c (**c**, **d**) expression in *M. tuberculosis* H37Ra overexpressing Rv3095. **a**, **c** Relative quantification by qPCR. Error bars indicate standard deviation. One-tailed Student’s *t*-tests were performed. **b**, **d** RNA-seq analysis. Error bars indicate standard deviation. Student’s *t*-tests were one-tailed. Control: H37Ra containing an empty pMV261 vector. *n* = 3 biologically independent experiments. *P* = <0.05 was considered significant. **e**–**g** Representative HPLC-UV spectra for *E. coli* BL21 harboring an empty pET28a vector incubated with 100 μg/ml ETH (**e**), *E. coli* BL21 harboring pET28a-Rv3094c in the absence of ETH (**f**), and *E. coli* BL21 harboring pET28a-Rv3094c incubated with 100 μg/ml ETH (**g**). *n* = 3 biologically independent experiments.

We first performed qPCR and RNA-Seq on an H37Ra strain overexpressing Rv3095 to confirm the regulatory relationship between Rv3095 and Rv3094c/Rv3093c. Rv3095 overexpression led to significant decreases in Rv3093c and Rv3094c expression, indicating that Rv3095 negatively modulates the expression of these genes (*p* < 0.05) (Fig. 4a–d; Supplementary Data 14).

We then investigated if putative oxygenase Rv3094c, like EthA, may be involved in ethionamide metabolism. Ethionamide activation by EthA^28^ is generally understood to occur in two steps; ethionamide (ETH) is first oxidized to its S-oxide (ETH-SO), and then forms a covalent adduct with NAD (ETH-NAD) that targets InhA as a tightly binding inhibitor^29^. As the activity of purified EthA is very low (k_cat_ = 0.0045 s^−1^)^30^, we chose to use an *E. coli* cell-based system to investigate the possible role of Rv3094c in ethionamide bioactivation rather than performing an in vitro biochemical characterization of purified Rv3094c enzymatic activity. *E. coli* does not have Rv3094c or *ethA* homologs and is not killed by ethionamide even at concentrations of 100 µg/ml, making it a suitable model system^30^. When we incubated *E. coli* BL21 Rv3094c or *ethA* overexpression strains and *E. coli* BL21 control strains with ethionamide, the ethionamide concentration (HPLC-UV retention time, 22.6 min) in supernatants from Rv3094c and *ethA* overexpression strains declined over time, and a new peak (molecular weight: 183.05, determined by MS) appeared at 27.1 min (Fig. 4e–g; Supplementary Fig. 3). We synthesized an ETH-SO standard, and confirmed the new peak to be ETH-SO using LC-MS/MS (Supplementary Fig. 3). We thus concluded that Rv3094c must be a monooxygenase that can directly sulfoxidise ethionamide, forming ETH-SO.

### Structure of Rv3094c and its complex with ethionamide

**Fig. 5:**
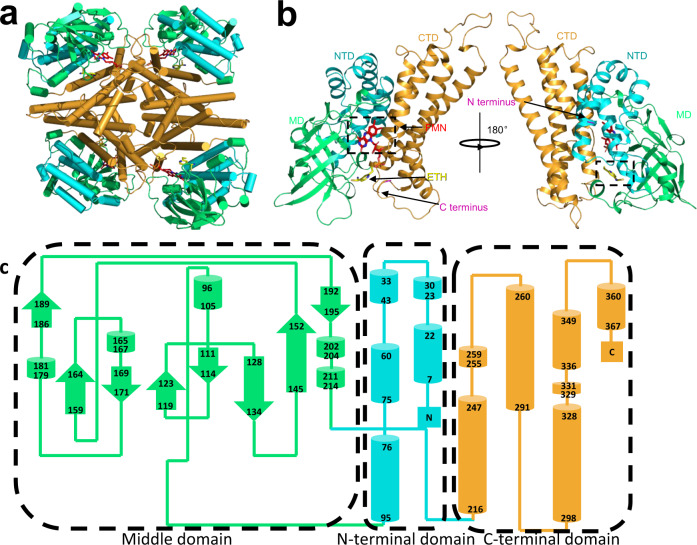
Structure of the Rv3094c-FMN-ETH complex. **a** Ribbon diagram of the Rv3094c-FMN-ETH tetramer. Cyan: N-terminal domain (residues 7–95); green: middle domain (96–214); orange: C-terminal domain (216–376). **b** Overall structure of the Rv3094c-FMN-ETH monomer, color-coded as defined in (**a**). **c** Topology diagrams of apo-Rv3094c. Cyan: N-terminal domain (residues 7–95); green: middle domain (96–214); orange: C-terminal domain (216–376).

Crystal structures of proteins shown to activate ethionamide (EthA, MymA, Rv0077c, and Rv0565c) have not yet been reported, making it difficult to determine the ethionamide-binding site and unravel the mechanistic details of ethionamide activation. To investigate the mechanism of ethionamide sulfoxygenation by Rv3094c, we solved protein crystal structures of apo-Rv3094c (Supplementary Fig. 4a), and Rv3094c in complex with FMN (Rv3094c-FMN; Supplementary Fig. 4b) and ethionamide (Rv3094c-FMN-ETH; Fig. 5a; Supplementary Figs. 4c and 5), at resolutions of 1.91 Å, 2.00 Å, and 1.64 Å, respectively. All three crystals contained one tetramer per asymmetric unit (Supplementary Fig. 4a, b), tetramers being formed by two homodimers, the C-terminal domain primarily mediating interactions between the two monomers (Supplementary Fig. 4d). The overall structure of the apo-Rv3094c monomer consists of an N-terminal domain (residues 7–95) composed of five α-helices, a middle domain (residues 96–214) composed of four α-helices and an eight-stranded β-barrel fold, and a C-terminal domain (residues 216–376), with a seven-helix bundle structure (Fig. 5b, c). The lack of a Rossmann fold suggests that Rv3049c can utilize either FAD or FMN, explaining the presence of FMN rather than FAD in the crystal structure obtained.

A structure-based comparison between Rv3094c, one-component monooxygenases, two-component monooxygenases, and Baeyer-Villiger monooxygenases (Supplementary Fig. 6) showed that Rv3094c clusters with two-component monooxygenases. As Rv3094c is co-transcribed with Rv3093c (Supplementary Data 11), a predicted FAD/FMN reductase, and FAD in ACAD family proteins is often reduced by an associated flavoprotein, we reasoned that Rv3093c and Rv3094c may form a two-component system in which Rv3094c is the monooxygenase partner responsible for the sulfoxygenation of ethionamide.

### Mechanism of Rv3094c sulfoxygenation of ethionamide

**Fig. 6:**
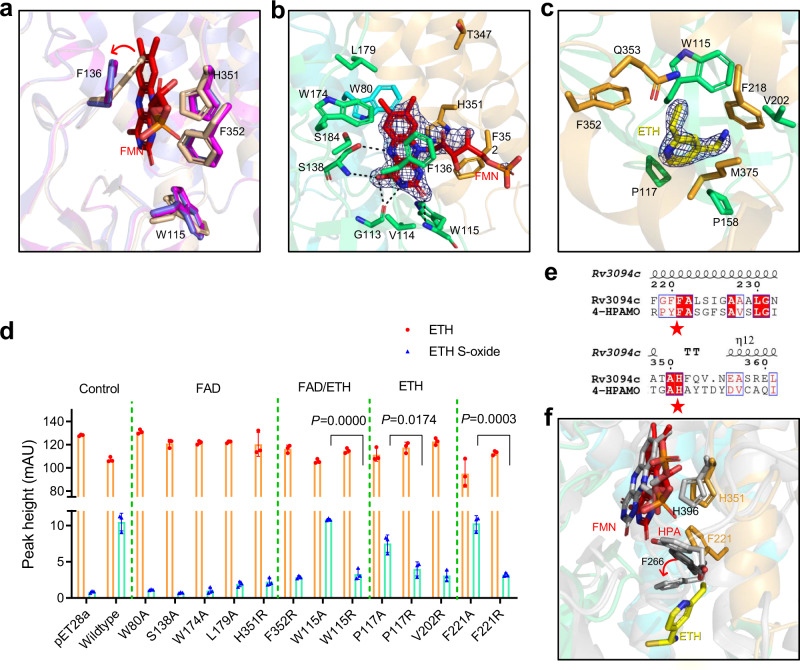
Structural analysis of the mechanism of Rv3094c-catalyzed activation of ethionamide. **a** Conformation of residues in the FMN-binding pocket. The four residues of the FMN binding pocket in apo-Rv3094c, Rv3094c-FMN, and Rv3094c-FMN-ETH are colored wheat, magenta, and slate, respectively. FMN is colored red. The red arrow indicates the swinging out of the F136 side chain to create the FMN pocket. **b** Interactions between FMN and Rv3094c, color-coded as defined in Fig. 5a. The color of each specific amino acid carbon is the same as its domain; the carbon of FMN is shown in red; nitrogen is shown in blue; oxygen is also shown in red. Hydrogen bonds are indicated by dashed lines. The 2Fo-Fc omit map is contoured at the 1.0 σ level. **c** Interactions between ethionamide and Rv3094c, color-coded as defined in (**b**), except that the carbon of ethionamide is shown in yellow. The 2Fo-Fc omit map is contoured at the 1.0 σ level. **d** Ethionamide-bioactivation activity of the Rv3094c wild-type protein and its mutants. HPLC-UV peak height was used as a measure of enzyme activity. Controls: empty pET28a vector (negative), wild type Rv3094c (positive). Dashed-green lines separate different groups of mutations: those of residues interacting with FAD and/or ethionamide. Residue F221 may act as a gate separating ethionamide from FMN. Student’s *t*-tests were unpaired, two-tailed. Error bars indicate standard deviation. *n* = 3 biologically independent experiments. **e** Sequence conservation analysis of Rv3094c and 4-HPAMO. The alignment of Rv3094c (NP_217610.1) and 4-HPAMO (4-HPA 3-monooxygenase large component, UniProtKB: Q6Q272.1) was generated using Clustal omega and the figure was prepared using ESPript (http://espript.ibcp.fr/ESPrip). The secondary structure of apo-Rv3094c is shown above the sequence. Strictly conserved residues are boxed in white on a red background and highly conserved residues are boxed in red on a white background. H396, and F226 in 4-HPAMO and its homologous residues in Rv3094c are marked with red stars. **f** Conformations of conserved substrate binding sites in Rv3094c and 4-HPA-hydroxylase, colored as defined in (**c**). Gray: 4-HPA-hydroxylase and HPA; F266 of 4-HPA-hydroxylase before HPA binding is shown in black.

Comparison of the above three structures indicates that FMN and ethionamide binding do not substantially alter the overall structure; the structure of Rv3094c-FMN-ETH is similar to that of apo-Rv3094c (Fig. 5, Supplementary Fig. 4a, b), having a root mean square deviation (RMSD) of 0.3 Å. Substrate binding, however, induces movements in the side chains of some amino acids in the apoenzyme and binary complex structures. F136 is the main residue involved in FMN binding pocket formation, and its side chain swings out to create the FMN binding pocket (Fig. 6a). The FMN flavin ring of FAD is held in position by a network of hydrogen bonds (involving G113, W115, and S138) and hydrophobic interactions (involving W80, V114, F136, W174, L179, S184, T347, H351, and F352) (Fig. 6b; Supplementary Data 15). Superposition of apo-Rv3094c structural homologs (Supplementary Fig. 7) not only reveals strong structural conservation of overall topology, but confirms that the location of the FMN binding site, lying in the pocket between the β-sheet and the C-terminal domain, and stabilization of the flavin ring by hydrogen bonds are also conserved. Ethionamide is embedded within a hydrophobic pocket comprised of the β-barrel domain and the C-terminal helix domain and is surrounded by the hydrophobic side chains of W115, P117, P158, V202, F218, F352, Q353, and M375 (Fig. 6c).

To evaluate which amino acids are important in interactions between Rv3094c and FAD and/or ethionamide, we constructed mutants based on the structure of the Rv3094c-FMN-ETH complex. ETH-SO synthesis was markedly altered by mutation of either of the two residues involved in both the FAD and ethionamide pockets (F352 and W115) or those specific to the FAD (W80, S138, W174, L179, and H351) or ethionamide (P117 and V202) pockets, indicating that these residues are all important in FAD/ethionamide binding (Fig. 6d). Mutation of W115 and P117 to arginine (R) (changing the hydrophobic side chain into a hydrophilic side chain with an additional 3-carbon aliphatic straight chain) blocked ethionamide activation more notably than when these residues were mutated to alanine (A) (simply removing the large side chain).

The first step in the reaction of reduced flavin with oxygen, as catalyzed by flavin-dependent monooxygenases, is the formation of flavin C4a-hydroperoxide which then acts as a nucleophile attacking the substrate^31^. In the structure of *p*-Hydroxyphenylacetate (HPA) hydroxylase, a structural and functional homolog of Rv3094c, HPA passes through a gate formed by F266 to interact with FMNH^-^ (Fig. 6e, f). Mutation of F221 (the F266 homolog) in Rv3094c to F221A or F221R (Fig. 6d), decreased ETH-SO synthesis, the F221A mutation resulting in a weaker steric effect than the wild-type F221 and only partial loss of the gating mechanism, whereas steric hindrance was increased in F221R, preventing the interaction between ethionamide and FAD and resulting in a more marked decrease in ethionamide hydroxylation. These findings indicate that F221 likely acts as a gate separating ethionamide from FMN, and has a role comparable to that of F266 in HPA hydrolase (Fig. 6f). In our structure, however, this FMN gate was closed, possibly because the structure we obtained was post-reaction rather than a reaction intermediate as we used FAD rather than FAD^-^ when preparing crystals of the complex.

## Discussion

Greater understanding of drug resistance mechanisms is important for combatting the increasing prevalence of drug resistance in *M. tuberculosis*. Our multi-omic systems-wide approach has yielded important insights; examining perturbations in gene/protein expression levels uncovered 68 drug resistance-associated gene pairs/clusters that may contribute to drug resistance in the 5–30% resistant strains (varying with antibiotic) with unknown resistance mechanisms^6^. In-depth investigation indicated that Rv3094c is likely a flavin-dependent monooxygenase that sulfoxygenates ethionamide, and that Rv3095 is a transcription regulator that regulates its expression. Using structures of apo-Rv3094c and its complex with FMN and ethionamide, the first reported crystal structures to the best of our knowledge of a protein involved in ethionamide activation, we identified key residues in its ethionamide and FMN binding pockets, and the gate through which ethionamide and FMN interact to complete the sulfoxidation reaction, shedding light on important mechanistic aspects of Rv3094c involvement in ethionamide metabolism.

The advent of powerful omic technologies has profoundly changed approaches to investigating drug resistance^32,33^. However, the large datasets generated require extensive bioinformatics analysis, and integrating data from different omic levels in bacterial systems is complicated; standard analytical pipelines that can be adapted for particular interests are still under development, and the order in which different omics data are analyzed and combined gives a different slant to analytical outcomes. Here, as our goal was to identify unknown genes/proteins associated with drug resistance mechanisms involving perturbations in gene expression, potentially revealing as yet undiscovered drug targets, our primary analysis was of resistance-associated variation in the transcriptome, genomic and proteomic data being integrated thereafter to hone in on genes/proteins of interest. Given that genes associated with particular functions/metabolic pathways are often arranged in pairs/clusters in bacterial genomes and transcribed as a single transcription unit^25^, we focused on adjacent gene pairs/clusters that were differentially-expressed rather than single genes. Unlike SNPs, the products of transcription are not fixed and are influenced by many extrinsic and intrinsic factors. By focusing on transcription, we have gained insight into a previously unknown set of potential drug resistance associations/mechanisms in which the perturbations in transcription products that lead to resistance are not directly related to genomic mutations.

The major genetic lineages of *M. tuberculosis* are strongly associated with specific geographical regions^15^. Reports suggest that early interactions between *M. tuberculosis* and its host are also determined by the lineage of the infecting strain; major modern lineage strains of *M. tuberculosis* exhibit distinct patterns of growth and cytokine induction^34^. Lineage is also correlated with inflammatory response; cytokine release from infected human peripheral blood monocyte-derived macrophages varies widely with the lineage of the infecting strain^35^. Our omics analysis indicated that expression data for 388 genes (Supplementary Data 6) has sufficient power to discriminate L2 and L4 isolates (Fig. 2c) suggesting that the expression of these genes may reflect lineage-specific characteristics of L2 and L4 isolates and lead to differences in growth, virulence, cytokine induction, and inflammatory phenotypes. After minimizing the effect of lineage on our analysis, we identified 68 drug resistance-associated gene pairs/clusters, including gene pairs *iniB-iniA*, *inhA-hemZ*, *embA-embB*, and *ethA-ethR*, whose resistance mechanisms are related to changes in gene expression^23,24^.

Interestingly, the gene structure of the Rv3094c-Rv3095 pair is similar to that of *ethA-ethR*, a putative monooxygenase/repressor pair involved in ethionamide metabolism. Rv3095 regulates the expression of Rv3093c-Rv3094c, and putative monooxygenase Rv3094c catalyzes the sulfoxygenation of ethionamide to ETH-SO (Fig. 4g), the initial step in its bioactivation. Current understanding of ethionamide bioactivation is that EthA, a flavoprotein containing a single FAD group, catalyzes NADPH- and O_2_-dependent monooxygenation of ethionamide to yield ETH-SO^36^. The ethionamide activation pathway is known to include redundancy; other ethionamide-activating proteins (MymA, Rv0077c, and Rv0565c) have been reported in recent years^37–39^. Given that overexpression of EthA in H37Ra in our MIC testing system led to considerably greater hypersensitivity to ethionamide than overexpression of Rv3094c (Supplementary Data 12), it is possible that Rv3094c plays a secondary role in ethionamide activation when EthA protein levels are high, however, the relationship between potential ethionamide-activating pathways is unclear and requires further elucidation. Our results indicate that Rv3094c can catalyze the initial step in ethionamide bioactivation, and that overexpression of Rv3094c in H37Ra (which also expresses *ethA*) increases phenotypic sensitivity to ethionamide 4-fold, indicating a role for Rv3094c in ethionamide metabolism and potentially in ethionamide resistance.

**Fig. 7:**
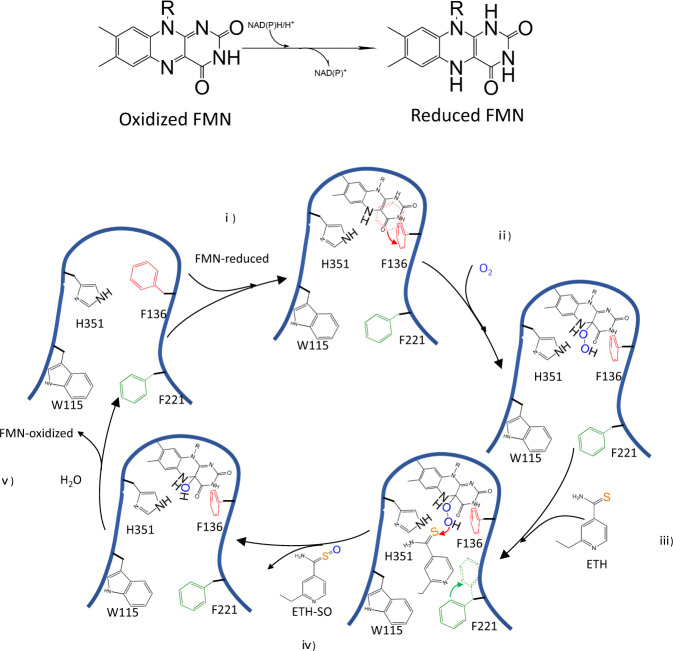
Model of catalytic events at the Rv3094c active site. Conversion of oxidized FMN to reduced FMN and schematic showing a model of the catalytic events that occur in the Rv3094c active site. (i) Conformational change occurs at F136, leading to Rv3094c binding of reduced FMN. (ii) An electron from reduced FMN then transfers to oxygen, forming the hydroperoxyflavin intermediate. During this process, H351 acts as a proton donor, stabilizing the hydroperoxyflavin intermediate. (iii) Opening of the gate between the flavin ring and ethionamde by conformational change in F221 allows ethionamide contact with hydroperoxyflavin and incorporation of an oxygen atom. Meanwhile, another oxygen atom is reduced, forming a water molecule. (iv) When the hydroperoxyflavin intermediate dissolves, the gate between the flavin ring and ethionamide closes, ethionamide sulfoxide is released, and (v) the oxidized FMN coenzyme within Rv3094c, together with NAD(P)H, starts a new cycle of ethionamide activation.

While other ethionamide-activating proteins (MymA, Rv0077c, or Rv0565c^37–39^) have been reported, to the best of our knowledge, there are no reported crystal structures of EthA, or indeed other Baeyer-Villiger monooxygenase family members or two-component flavin-dependent monooxygenases that contain an S-containing substrate and demonstrate S-oxidation. There are also few crystal structures of monooxygenases that oxidize sulfide among the 1332 reported monooxygenase structures. Lack of such crystal structures has made it difficult to determine the ethionamide-binding site and unravel the mechanistic details of ethionamide activation. Here, having compared our crystal structures of apo-Rv3094c (1.91 Å), and its Rv3094c-FMN (2.00 Å) and Rv3094c-FMN-ETH (1.64 Å) complexes, and performed related mutagenesis experiments, we identified key residues in the FMN and ethionamide binding pockets, and their interaction site. These findings, together with additional comparisons with the structure of *p*-Hydroxyphenylacetate (HPA) hydroxylase (PBD ID: 2JBT), a structural and functional homolog of Rv3094c, in complex with HPA, and its predicted bioactivation mechanism, have led us to postulate a mechanism by which ethionamide is sulfoxygenated to ETH-SO by Rv3094c (Fig. 7): oxidized FMN is reduced by an as yet unidentified enzyme containing a NAD(P)H cofactor. (i) A conformational change occurs in F136 and Rv3094c binds reduced FMN. (ii) An electron from reduced FMN then transfers to oxygen, forming a hydroperoxyflavin intermediate. During this process, H351 acts as a proton donor, stabilizing the hydroperoxyflavin intermediate. (iii) Conformational change in F221 then opens the gate between the flavin ring and ethionamide, allowing ethionamide contact with hydroperoxyflavin and incorporation of an oxygen atom. Another oxygen atom is reduced, forming a water molecule. (iv) When the hydroperoxyflavin intermediate dissolves, the gate between the flavin ring and ethionamide closes, ETH-SO is released, and (v) the oxidized FMN coenzyme within Rv3094c, together with NAD(P)H, starts a new cycle of ethionamide activation.

Our study has a number of limitations: (1) We could only include 11 *M. tuberculosis* isolates in this study, and could only investigate one potential resistance-associated gene pair/cluster in depth. The drug resistance associations detected here will need to be verified in a broader collection of clinical isolates to confirm their clinical relevance. (2) The transcriptomic/proteomic data analyzed was collected in the absence of drug stimulation so we could hone in on native physiological and biochemical mechanisms/states that underlie drug resistance. Our study, therefore, does not address drug-induced changes in expression that may lead to phenotypic resistance in intrinsically drug sensitive isolates^23,24,40^. Emerging evidence that drug-induced proteomic changes can lead to drug tolerance/resistance in drug sensitive isolates is an important consideration that requires further investigation, particularly given the growing momentum in the field for widespread application of molecular testing. (3) We did not perform in vitro validation of the ethionamide sulfoxidation activity of Rv3094c detected in vivo (Fig. 4g and Supplementary Fig. 3) using purified Rv3094c protein. Future biochemical characterizations of Rv3094c activity and comparisons with other known ethionamide-activating proteins (EthA, MymA, Rv0077c, or Rv0565c) will provide further insights into the respective involvement of these proteins in ethionamide activation and mechanisms leading to ethionamide resistance.

Our integrated multi-omic study of 11 *M. tuberculosis* isolates varying in lineage (L2 and L4) and drug resistance profile has demonstrated that lineage-related transcriptomic and proteomic variation reflects phylogenetic relationships, and has identified drug resistance associations involving perturbations in the expression of specific gene pairs/clusters that may merit further investigation. Studies on the Rv3093c-Rv3095 gene cluster suggest that Rv3094c may be involved in ethionamide metabolism, and mutagenesis studies based on the first crystal structures (to the best of our knowledge) of an ethionamide-sulfoxygenating protein, identified key aspects of its ethionamide-sulfoxygenation mechanism. Our findings suggest that integrated multi-omic strategies combining genomic, transcriptomic, and proteomic data have potential to greatly enhance our understanding of drug resistance mechanisms and lineage-related pathogenic characteristics of *M. tuberculosis*.

## Methods

### Ethics approval

Clinical isolates collection was approved by the Ethics Committee of Chinese Center for Disease Control and Prevention, Beijing. Isolates were provided by State Key Laboratory for Infectious Diseases Prevention and Control, National Institute for Communicable Disease Control and Prevention, Chinese Center for Disease Control and Prevention.

### Bacterial strains and growth conditions

*Mycobacterium tuberculosis* isolates from patients with active TB disease were obtained from the Chinese Center for Disease Control and Prevention (Beijing, China). Eleven isolates (4 drug-sensitive isolates, 4 multi-drug-resistant isolates, and 3 extensively drug-resistant isolates; Supplementary Data 1) were selected after testing for drug sensitivity. All strains were grown on Lowenstein-Jensen slants or Middlebrook 7H9 broth containing 10% OADC (an oleate–albumin–dextrose–catalase (OADC) supplement (Becton Dickinson) at 37 °C for 4–6 weeks to reach log phase.

### Drug sensitivity testing

The 11 isolates were tested for drug susceptibility on solid media using the standard proportion method recommended by the World Health Organization^2^. The following drug concentrations were used: isoniazid (INH), 0.2 μg/ml; streptomycin (STR), 4.0 μg/ml; rifampicin (RFP), ethionamide (ETH), and capreomycin (CPM) 40.0 μg/ml; ethambutol (EMB) and ofloxacin (OFX), 2.0 μg/ml; kanamycin (KAN), 30.0 μg/ml.

### Genomics

DNA sequencing data for previously sequenced isolates S01 (H37Rv), S03 (SH045), S04 (SH396), R01 (FJ05120), R02 (FJ05132), R03 (FJ05195), R04 (FJ07070), R05 (SH400), R06 (XZ06030), and R07 (XZ06217)^20^ was obtained from NCBI Sequence Read Archive (SRA) accession SRA065095. Isolate S02 (GZ10057) was sequenced de novo. Genomic DNA was extracted from cells cultured under uniform conditions for 4–6 weeks using CTAB (cetyltrimethylammonium bromide) according to a standard protocol^41^. Sequencing data was obtained using the same method as used previously for the other isolates^20^. Sequencing reads for isolate S01 (GZ10057) have been submitted to NODE (https://www.biosino.org/node/index) under accession OER036225.

### Transcriptome sequencing

Total RNA samples were prepared from three biological replicates per isolate. Total RNA was extracted from logarithmic phase cells cultured under uniform conditions for 4–6 weeks using TRIzol^®^ (Invitrogen Life Technologies)^42,43^, resuspended in RNase-free water and stored at −80 °C. Bacterial mRNA was fragmented and converted into a strand-specific RNA-seq library using the KAPA Stranded RNA-Seq kit (KAPA Biosystems, USA) according to the manufacturer’s instructions. For both cDNA and genomic libraries, cluster generation was performed on an Illumina C-bot (Illumina, San Diego, CA, USA) and 2 × 150 bp paired-end sequencing was performed using an Illumina HiSeq X10 (Illumina, San Diego, CA, USA). A total of 119 Gb of 2 × 150 bp data was obtained with an average Q30 > 97.4% and each sample was represented by at least 32 million pairs of reads^44^. Data were first examined for quality using Fastqc (http://www.bioinformatics.babraham.ac.uk/projects/fastqc). Reads of length <35 and quality score >20 were filtered out using Trimmomatic-0.36^45^. Filtered reads were mapped to the H37Rv reference genome (NC_000962) with STAR^46^. FKPM values were calculated using featureCount^47^ based on read counts of the corresponding genes obtained with multicov from the Bedtools suite (https://bedtools.readthedocs.io/en/latest/content/tools/multicov.html).

### Sample preparation for proteomic analysis

Total protein was extracted from logarithmic phase cells (one sample per isolate). Cells were washed three times with chilled phosphate buffer saline (PBS), lysed in lysis buffer (9 M Urea, 10 mM Tris-HCl (pH 8.0), 30 mM NaCl, 50 mM IAA, 5 mM Na_4_P_2_O_7_, 100 mM Na_2_HPO_4_, 1 mM NaF, 1 mM Na_3_VO_4_, 1 mM sodium glycerophosphate, 1% phosphatase inhibitor cocktail 2 (Sigma, St. Louis, USA), 1% phosphatase inhibitor cocktail 3 (Sigma, St. Louis, USA), EDTA-free protease inhibitor cocktail (1 tablet/10 ml lysis buffer, Roche, Basel, Switzerland)^48^, then centrifuged at 13,000 rpm for 10 min at 4 °C to remove cellular debris. All samples were stored at −80 °C. Quantification of protein concentration and in-gel digestion were performed as previously described^49^. In brief, 100 μg of each total protein sample was reduced with dithiothreitol (5 mM), alkylated with iodoacetamide (20 mM), precleaned with 10% SDS-PAGE (0.7 cm), and digested in-gel with trypsin (12.5 ng/μl; Meizhiyuan, Beijing, China) at 37 °C for 12 h^50^. Tryptic peptides were labeled with TMT reagents according to the manufacturer’s instructions (Thermo Scientific, Rockford, USA). Samples were divided into two groups, S01, S02, S03, R01-1, R01-2, R02, R03, R04, R05, and R07 (Group 1) being labeled with 127^C^, 131, 130^C^, 128^N^, 129^C^, 130^N^, 128^C^, 127^N^, 129^N^, and 126, respectively, and S01-1, S01-2, S04, and R06 (Group 2) being labeled with 126, 127^N^, 130^N^, and 128^C^, respectively, as only 8 samples could be processed at one time. Labeling efficiency was ≥95%. The labeled samples in each group were combined, and dried under vacuum^51^.

### One-dimensional separation of TMT-labeled peptides

Mixed TMT-labeled samples were separated on a one-dimensional high pH reverse phase (RP) HPLC system (Rigol, L-3120, Beijing, China) as described previously^49^. Each mixed sample was dissolved in 400 µl buffer A (98% ddH_2_O and 2% ACN, pH 10, adjusted with ammonium hydroxide), injected into a Durashell C_18_ column (150 Å, 5 μm, 4.6 × 250 mm^2^) and eluted with a linear gradient over a period of 60 min using buffer B (2% ddH_2_O and 98% ACN, pH 10; 0% B for 5 min, 0–3% B for 3 min, 3–22% B for 37 min, 22–32% B for 10 min, 32–90% B for 1 min, 90% B for 2 min, and 100% B for 2 min). The LC flow rate was set at 0.7 ml/min and monitored at 214 nm. The column was maintained at 45 °C. Sixty fractions were collected and after the first 10 were discarded, the remaining fractions were concatenated into 8 mixed fractions by combining fractions 11, 19, 27, 35, 43, 51, 59; fractions 12, 20, 38, 35, 43, 51, 60; and so on, according to peak capacity. In order to analyze samples simultaneously, Group 1 and 2 samples were analyzed on separate mass spectrometers.

### LC-MS/MS of TMT-labeled peptides

Sample group 1: TMT-labeled peptides from each fraction were first separated on an Easy-Nano LC 1200 (ThermoFisher Scientific, Waltham, MA, USA), then analyzed on an Orbitrap Fusion Lumos mass spectrometer (ThermoFisher Scientific, Waltham, MA, USA). Briefly, samples were loaded onto a self-packed capillary column (75 μm i.d. × 50 cm, 1.9 μm C_18_) and eluted with a 90 min linear gradient (from 1 to 40% buffer B (Buffer A: 5 mM ammonium formate in 2% ACN; Buffer B: 5 mM ammonium formate in 90% ACN). Full MS scans were performed with *m*/*z* ranges of 375–1500 at a resolution of 1.2 × 10^5^; the maximum injection time (MIT) was 50 ms, and the automatic gain control (AGC) was set to 4 × 10^5^. For MS/MS scans, the 20 most intense peptide ions with charge states of 2 to 7 were subjected to fragmentation via higher energy collision-induced dissociation (HCD) (AGC: 5 × 10^4^, MIT: 118 ms, Resolution: 6 × 10^4^). Dynamic exclusion was set as 15 s.

Sample group 2: TMT-labeled peptides from each fraction were first separated on an Easy-Nano LC 1200 (ThermoFisher Scientific, Waltham, MA, USA), then analyzed on a Q Exactive HF mass spectrometer (ThermoFisher Scientific, Waltham, MA, USA). Briefly, samples were loaded onto a self-packed capillary column (75 μm i.d. × 50 cm, 1.9 μm C_18_) and eluted with a 135 min linear gradient (4–8% B for 13 min, 8–25% B for 86 min, 20–50% B for 21 min, 50–90% B for 3 min, 90% B for 12 min). Full MS scans were performed with *m*/*z* ranges of 375–1400 at a resolution of 1.2 × 10^5^; the maximum injection time (MIT) was 80 ms, and the automatic gain control (AGC) was set to 3.0 × 10^6^. For the MS/MS scans, the 15 most intense peptide ions with charge states of 2 to 6 were subjected to fragmentation via higher energy collision-induced dissociation (HCD) (AGC: 1 × 10^5^, MIT: 100 ms, Resolution: 6 × 10^4^). Dynamic exclusion was set as 30 s.

Raw files from each group were searched against *M. tuberculosis* databases using software provided with each MS instrument; Group 1 raw files were searched against the Uniprot reference proteome from the *M. tuberculosis* H37Rv database with Proteome Discoverer (v2.1.1.21)^52^, and Group 2 raw files were searched with MaxQuant (v1.5.6.0)^53^ against the equivalent TubercuList database. Proteins were identified based on the presence of at least two matching peptides. Two missing tryptic peptides were allowed. Oxidation of methionine was set as a dynamic modification, and carbamidomethylation of cysteine, and TMT modification at both the peptide N-terminus and lysine were set as static modifications. The tolerance of the precursor and fragment ions was set to 20 ppm.

### Multi-omic analysis—lineage-associated variation

Lineage-related SNPs were defined as those that have the same allele within a given lineage but different alleles between lineages. Genomic data for each lineage 2 and 4 isolate was compared separately with the H37Rv reference genome (NC_000962) to identify lineage-specific SNPs. Differentially-expressed genes (Log foldchange >1 or <−1, *p*-value <0.05) were determined by comparing the transcriptomes of respective L2 and L4 isolates using the R package edgeR^54^. Differentially-expressed proteins were defined as those whose expression varied more than two-fold in one-to-one comparisons of quantitative mass spectrometry data between respective L2 and L4 isolates from the same group. In Group 1, S01, R01 and R02 were compared with S02, S03, R03, R04 R05, and R07, and in Group 2, S01 and R06 were compared with S04. The number of times a protein was differentially expressed in these one-to-one comparisons was recorded.

### Multi-omic analysis—DR-associated variation

To minimize the influence of lineage in the analysis of drug resistance-associated variation, we performed one-to-one comparisons of drug sensitive and drug resistant isolates from within the same lineage (L2: R03, R04, R05, R07, S02, S03, S04; L4: S01, R01, R02, and R06). Drug resistance-associated SNPs were identified by comparing genomic data for each drug resistant and drug sensitive isolate. Differentially-expressed genes (Log foldchange >1 or <−1, *p*-value <0.05) were determined by comparing the transcriptomes of each drug resistant and drug sensitive isolate using the R package edgeR^54^. Differentially-expressed proteins were defined as those with log_2_ (fold change) >1 or <−1 expression changes in one-to-one comparisons of quantitative mass spectrometry data between respective drug resistant and drug sensitive isolates from the same group (*p* < 0.05). In Group 1, S01 was compared with R01 and R02, and S02 and S03 were compared with R03, R04, R05, and R07, while in Group 2, S01 was compared with R06.

Genes located adjacently as pairs or clusters in the genome that simultaneously showed differential gene expression changes (upregulation or downregulation) in at least one of the drug resistant: drug sensitive isolate comparisons, and at least one of the genes either showed differential protein expression (upregulation or downregulation) or contained drug resistance-associated SNPs, were considered as drug resistance-associated gene pairs/clusters.

### qRT-PCR and transcriptomic analysis of Rv3093c and Rv3094c regulation

Total RNA was extracted from three independent cultures of recombinant *M. tuberculosis* H37Ra harboring pMV261-Rv3095 or an empty pMV261 plasmid grown to early log phase on Middlebrook 7H10 agar supplemented with 10% OADC (BBL Microbiology Systems, Cockeysville, MD, USA) using a Bacteria RNA Extraction Kit (Vazyme Biotech, Nanjing, China). Complementary DNA (cDNA) was synthesized using the SuperScript III^®^ First-Strand Synthesis System (Invitrogen), according to the manufacturer’s instructions, and quantitative real-time PCR of Rv3093c, Rv3094c, and Rv3095 was performed according to a previously published method^55^. Briefly, qRT-PCR was performed using a QuantiNovaTM SYBR® Green PCR Kit (QIAGEN, Hilden, Germany) on a ABI 7500 system (Applied BioSystems, Foster City, USA), each reaction having a final volume of 20 μl and comprising 10 μl 2 × SYBR Green PCR Master Mix, 2 μl cDNA, 1 μl of each primer (10 μM), 0.1 μl QN ROX Reference Dye and RNase-free water. Reaction conditions: PCR initial heat activation at 95 °C for 2 min, 40 cycles of 95 °C for 5 s and 60 °C for 30 s. The relative quantity of target gene mRNA was determined by reference to mRNA levels of the *M. tuberculosis* housekeeping gene *sigA*, calculated as:

1 $${{{{{\rm{Fold}}}}}}\,{{{{{\rm{change}}}}}} = 	 \, {2}^{-\varDelta (\varDelta Ct)},\,{{{{{\rm{where}}}}}}\,\varDelta Ct=C{t}_{({{{{{\rm{target}}}}}})}-C{t}_{(sigA)},\\ {{{{{\rm{and}}}}}}\,\varDelta (\varDelta Ct) =	 \, \varDelta C{t}_{({{{{{\rm{overexpression}}}}}})}-\varDelta C{t}_{({{{{{\rm{control}}}}}})}$$

Primers used for qRT-PCR analysis were: *Rv3093c*-qF: CAGTTCGGTCGCTTCTCTCA; *Rv3093c*-qR: CATTCGATGGTTTCGCGCAT; *Rv3094c*-qF: AGCATGTATGATCTGGCGGG; *Rv3094c*-qR: GATGCCTCGTTGACCTGGAA; *Rv3095*-qF: TTGACCGGGTGCCATATTCC; *Rv3095*-qR: CAGCTTGATCGGTGGTCCTT; *SigA*-F: CGATGACGACGAGGAGATCGC; and *SigA*-R: CAGCGCTACCTTGCCGATCTG. RNA samples of the two strains (3 biological replicates) were also submitted for transcriptome profiling following the transcriptomics methods outlined above.

### Ethionamide bioactivation assay

Rv3094c and *ethA* were amplified from purified *M. tuberculosis* H37Rv genomic DNA. PCR products were ligated into pET-28a (Novagen), generating plasmids encoding the corresponding proteins with a 6×His tag at their N-terminus. Plasmids pET28a-Rv3094c, pET28a-*ethA,* and empty pET28a were then, respectively, transformed into *E. coli* BL21(DE3) cells and grown to an OD_600_ of 0.6–0.8 in LB liquid medium. Protein expression was induced by addition of isopropyl β-D-thiogalactopyranoside (IPTG; final concentration: 0.2 mM) and proceeded for 16 h at 16 °C. Cultures were then incubated with/without ethionamide (final concentration: 80 µg/ml) for 6 h at 37 °C. All experiments were performed in triplicate.

Ethionamide concentration and metabolite identification were determined at 0, 3, and 6 h after addition of ethionamide (except for cultures of *E. coli* BL21 (DE3) harboring an empty pET28a vector in the absence of ethionamide). Samples were centrifuged at 12,000 rpm for 10 min, and supernatants were filtered (0.22 μm; Sangong, China) before analyzing on a Dionex UltiMate 3000 HPLC system (Sunnyvale, CA, USA) equipped with an UltiMate 3000 pump, UltiMate 3000 autosampler column compartment and UltiMate 3000 VWD Variable Wavelength Detector. Data acquisition was performed with Chromeleon software (Sunnyvale, CA, USA). Chromatographic separation was performed as previously described^36^. Briefly, experiments were performed on a reversed phase AcclaimTM120 C8 column (5 μm, 4.6 mm × 250 mm, Thermo Scientific, Sunnyvale, USA). The column was maintained at 40 °C throughout the analysis and a wavelength of 284 nm was used for detection. The mobile phases consisted of 0.01% formic acid in water (A) and 0.01% formic acid in acetonitrile (B). The mixture was eluted with a gradient of 1–8.3% B over a period of 30 min.

### Minimum inhibition concentration (MIC) determination

The tested genes were amplified from *M. tuberculosis* H37Rv genomic DNA and, respectively, ligated into pMV261 vectors at the *Bam*HI and *Hin*dIII restriction sites using Hieff Clone^®^ Plus One Step Cloning Kits (Yeasen, China), before transforming into *M. tuberculosis* H37Ra according to standard methods^56^. The recombinant *M tuberculosis* H37Ra strain was characterized genetically by PCR analysis. A primer pair targeting pMV261 (pMV261-f, 5’-CAACAGCATTTGGCGTTGGA-3’ and pMV261-r, 5’-TTTACCTGTCCGACCCGTTG-3’) was used for PCR analysis, yielding a 596 bp product. PCR reactions were performed in a 20 µl reaction volume containing 10 µl 2× Taq mixture, 2 µl genomic DNA template, and 0.5 µmol of each primer set. PCR conditions: 94 °C denaturation for 5 min, followed by 30 cycles of 94 °C denaturation for 1 min, annealing at 60 °C for 1 min, and extension at 72 °C for 1 min, with a final extension cycle at 72 °C for 7 min. 5 µl of the PCR product obtained was analyzed by running on a 1.5% agarose gel. MIC determination against RIF, INH, STR, EMB, OFX, and CPM was performed using a modified resazurin microtiter assay^57^. Briefly, serial two-fold dilutions of each drug were prepared directly in 96-well plates at the following concentrations: RIF: 0.001 to 1.024 µg/ml, INH: 0.0125 to 12.8 µg/ml, STR, EMB, OFX, and CPM: 0.0625 to 64 µg/ml. Inoculum of the recombinant *M tuberculosis* H37Ra strains carrying the gene to be tested were prepared from fresh Middlebrook 7H10 slants in 7H9 medium containing 10% OADC, adjusted to a McFarland density of 1.0, then diluted 1:10. 100 µl of the inoculum was added to each well and the plate was covered, sealed with parafilm, and incubated at 37 °C. After 7 days of incubation, 20 µl of resazurin solution (filter sterilized, 10 mg/ml concentration) was added to each well, and the plate was incubated for 24–48 h. A change in color from blue to pink indicated the growth of bacteria, and the MIC was defined as the lowest concentration of drug that prevented this change in color.

MIC determination against ethionamide was performed using customized ethionamide MIC microplates (ethionamide concentrations of 0.0625 to 64 µg/ml in serial two-fold dilutions; Baso Diagnostics, Zhuhai, China). The inoculum was prepared as described above, but was diluted 1:100. 100 µl inoculum was added to each well and microplates were covered, sealed in plastic bags, and incubated at 37 °C for 7 to 8 days. Microplates were read directly using an inverted mirror to detect growth in the wells. The MIC was defined as the lowest concentration of ethionamide that inhibited the visible growth of the bacterium.

### Rv3094c structure determination

The Rv3094c gene was amplified from *M. tuberculosis* H37Rv genomic DNA, ligated into a pET-28a-SUMO plasmid, and expressed in *E. coli* Rosetta (DE3) as a SUMO fusion protein. Cells were cultured to an OD_600_ of 0.6–0.8 in LB medium supplemented with 50 μg/ml kanamycin at 37 °C (220 rpm shaking). Protein expression was induced by adding 0.3 mM IPTG to the medium and culturing for a further 16 h at 18 °C. After centrifugation at 4500 × *g* for 15 min, cells were lysed by sonication in cold lysis buffer (50 mM Tris-HCl pH 8.0, 150 mM NaCl, 1 mM PMSF, 4 mM β-mercaptoethanol) and centrifuged twice at 18,000 × *g* (4 °C) for 30 min to remove bacterial debris. The supernatant was loaded onto a column containing 2 ml pre-equilibrated Ni-NTA Agarose (QIAGEN) and washed with 20 column volumes of binding buffer (50 mM Tris-HCl pH 8.0, 150 mM NaCl, 20 mM imidazole, 1 mM PMSF, 4 mM β-Mercaptoethanol). The His-SUMO tag was removed by addition of ULP1 protease at 4 °C for 12 h on the column and then the cleaved product was eluted in binding buffer. Rv3094c protein was then loaded onto a Q column (GE Healthcare), eluted with a NaCl gradient, then further purified using a Superdex200 column (GE Healthcare) in buffer containing 25 mM Tris-HCl pH 8.0, 200 mM NaCl, 2 mM DTT. The purified protein was flash-frozen in liquid nitrogen and stored at −80 °C.

The sitting-drop vapor diffusion method was used for initial crystallization screens of native Rv3094c. Crystallization drops contained 0.5 μl of protein solution mixed with 0.5 μl of reservoir solution. Rv3094c crystals were grown in the presence of 0.2 M sodium nitrate, 0.1 M Bis Tris propane pH = 8.5, 20% w/v PEG3350. Diffraction quality crystals were optimized under the same condition using the hanging drop (1 μl × 1 μl) vapor diffusion method at 16 °C. Rv3094c-FMN crystals and Rv3094c-FMN-ETH crystals were grown under the same condition by soaking in reservoir solutions containing 2 mM FAD and 2 mM FAD, 2 mM ethionamide, respectively. Crystals were cryoprotected in reservoir solution supplemented with 20% glycerol and flash-cooled by plunging into liquid nitrogen.

Data were collected at the Shanghai Synchrotron Radiation Facility using beamline BL17U (with X-rays at a wavelength of 0.97941 Å) and were processed and scaled with the HKL2000 suite^58^. The structure of Rv3094c was determined by molecular replacement with MOLREP in the CCP4 suite using the structure of flavin-dependent bacterial indoloterpenoid cyclase (PDB ID: 5MR6) as the search model^59,60^. The structures of Rv3094c-FMN and Rv3094c-FMN-ETH were determined by molecular replacement with the apo structure of Rv3094c as the search model. Refinement was performed with PHENIX^61^ and manual revision of the model was performed using Coot^62^. The quality of the final models was checked using the wwPDB Validation Service (https://validate-rcsb-1.wwpdb.org/validservice/). Structures were analyzed for their multimeric state using PDBePISA (https://www.ebi.ac.uk/pdbe/pisa/). 3D structures of homologous proteins were compared using Dali (http://ekhidna2.biocenter.helsinki.fi/dali/). All structural figures were made using PyMOL (http://pymol.sourceforge.net). Data collection and refinement statistics are summarized in Table 1.

**Table 1:** X-ray crystallography data collection and refinement statistics for Rv3094c.

Dataset	apo-Rv3094c	Rv3094c:FMN	Rv3094c:FMN:ETH
*Data collection*			
Beamline	BL-17U1, SSRF	BL-17U1, SSRF	BL-17U1, SSRF
Wavelength (Å)	0.9792	0.9792	0.9792
Space group	*I* 4_1_ 2 2	C 1 2 1	*I* 4_1_ 2 2
Cell dimensions			
* a*, *b*, *c* (Å)	133.58, 133.58, 109.69	172.06, 134.22, 109.41	134.10, 134.10, 109.59
* α*, *β*, *γ* (^o^)	90.00, 90.00, 90.00	90.00, 128.93, 90.00	90.00, 90.00, 90.00
Resolution range (Å)^a^	94.46–1.96 (8.54–1.91)	85.11–2.11 (6.32–2.00)	35.25–1.68 (7.33–1.64)
Completeness (%)	99.7 (100.0)	97.2 (95.7)	97.7 (100.0)
*I/σ(I)*	66.9 (2.4)	16.9 (2.7)	44.0 (2.2)
*R*_merge_	0.042 (1.636)	0.051 (0.406)	0.077 (1.645)
Multiplicity	20.9 (26.9)	3.2 (3.3)	15.3 (26.1)
CC half	1.000 (0.741)	0.992 (0.903)	0.985 (0.799)
Anomalous completeness (%)	100 (100)	91.7 (79.5)	95.9 (100.0)
Anomalous multiplicity	12.8 (13.8)	1.7 (1.7)	9.2 (13.4)
*Refinement*			
Resolution (Å)	1.91	2.00	1.64
No. reflections	36,751	123,410	60,943
*R*_work_/*R*_free_ (%)	19.62/23.04	19.43/22.61	17.74/19.93
No. atoms			
Protein	2750	11178	2785
Ligands		124	42
Solvent	190	676	140
*B*-factors (Å^2^)			
Protein	41.2	37.4	41.8
Ligands		49.9	41.5
Solvent	43.8	38.0	42
R.m.s. deviations			
Bond length (Å)	0.0064	0.011	0.0104
Bond angles (°)	0.78	1.09	1.14
Ramachandran plot (%)			
Favored region	97.58		
Allowed region	1.11	2.78	1.90
Outliers region	0.00	0.07	0.00

^a^Highest resolution shell is shown in parentheses.

### Statistics and reproducibility

Eleven *M. tuberculosis* isolates were included in this study, and could only investigate one potential resistance-associated gene pair/cluster in depth. The drug resistance associations detected here will need to be verified in a broader collection of clinical isolates to confirm their clinical relevance.

Student’s unpaired t-tests were used for comparisons of data derived from qPCR, RNA-seq, and ethionamide-bioactivation activity testing. The number of biological replicates, described as *n* = X, is indicated in the respective figure legend.

### Reporting summary

Further information on research design is available in the Nature Portfolio Reporting Summary linked to this article.

## Supplementary information

Supplementary Information

Description of Additional Supplementary Files

Supplementary Data 1-15

Reporting Summary

## Data Availability

Sequencing reads for isolate S01 (GZ10057) are available at the NODE repository (https://www.biosino.org/node/index) under accession OER036225. Other datasets generated and/or analyzed during the current study are available in NODE under accession codes OER036186-OER036224. DNA sequencing data for previously sequenced isolates S01 (H37Rv), S03 (SH045), S04 (SH396), R01 (FJ05120), R02 (FJ05132), R03 (FJ05195), R04 (FJ07070), R05 (SH400), R06 (XZ06030), and R07 (XZ06217) was obtained from NCBI Sequence Read Archive (SRA) accession SRA065095. Proteome MS data is deposited with the ProteomeXchange Consortium (http://proteomecentral.proteomexchange.org) under the identifier PXD017079. Protein structure data is deposited in the Protein Data Bank (PDB) (apo-Rv3094c: 7F70; Rv3094c-FMN: 7F74; Rv3094c-FMN-ETH: 7F72). Source data underlying figures are available in Supplementary Data 13 (Fig. 3c), Supplementary Data 14 (Fig. 4a–d), and Supplementary Data 15 (Fig. 6d). Any other associated data is available from the corresponding authors upon reasonable request.
