# Supplementary material for: Omics analysis of Mycobacterium tuberculosis isolates uncovers Rv3094c, an ethionamide metabolism-associated gene
Source: Commun Biol. 2023 Feb 7;6:156. doi: 10.1038/s42003-023-04433-w (PMC9904262; doi:10.1038/s42003-023-04433-w)
Supplement: Supplementary file 1 — Supplementary Information [file 42003_2023_4433_MOESM1_ESM.pdf]

Supplementary Information

Omics analysis of *Mycobacterium tuberculosis* isolates uncovers Rv3094c, an ethionamide metabolism-associated gene

Li Wan[1,2,3,4,†], Peilei Hu[1,5,6,†], Lili Zhang[1,6,†], Zhao-Xi Wang[2,†], Joy Fleming[1], Bo Ni[5], Jianjun Luo[1], Cha-Xiang Guan[4], Liqiong Bai[5], Yunhong Tan[5], Haican Liu[3], Na Li[3], Tongyang Xiao[3], Hua Bai[5], Yong-An Zhang[2], Xian-En Zhang[7], Kanglin Wan[3,*], Lijun Bi[1,8,*], Songying Ouyang[2,*] & Hongtai Zhang[1,9*]

[1] Key Laboratory of RNA Biology, Institute of Biophysics, Chinese Academy of Sciences, Beijing 100101, China

[2] Provincial University Key Laboratory of Cellular Stress Response and Metabolic Regulation, the Key Laboratory of Innate Immune Biology of Fujian Province, Biomedical Research Center of South China, Key Laboratory of OptoElectronic Science and Technology for Medicine of the Ministry of Education, Fujian Key Laboratory of Special Marine Bio-resources Sustainable Utilization, College of Life Sciences, Fujian Normal University, Fuzhou, 350117, Fujian Province, China.

[3] State Key Laboratory for Infectious Diseases Prevention and Control, Collaborative Innovation Center for Diagnosis and Treatment of Infectious Diseases, National Institute for Communicable Disease Control and Prevention, Chinese Center for Disease Control and Prevention, Beijing 102206, China

[4] Department of Physiology, Xiangya School of Medicine, Central South University, Changsha, Hunan 410078, China

[5] Hunan Chest Hospital, Changsha 410013, Hunan Province, China

[6] University of Chinese Academy of Sciences, Beijing 100049, China

[7] National Laboratory of Biomacromolecules, Institute of Biophysics, Chinese Academy of Sciences, Beijing 100101, China

[8] Guangdong Province Key Laboratory of TB Systems Biology and Translational Medicine, Foshan 528000, Guangdong Province, China

[9] Institute of Food Science and Technology, Chinese Academy of Agricultural Sciences, Beijing 100193, China

[†] Li Wan, Peilei Hu, Lili Zhang and Zhaoxi Wang contributed equally to this work

*Correspondence: wankanglin@icdc.cn, blj@ibp.ac.cn, ouyangsy@fjnu.edu.cn and hongtaizhang@aliyun.com

Table of contents

Table of Contents **2**

Supplementary Figures

Figure S1. KEGG and GO function analysis of 172 of the 388 *M. tuberculosis* genes expressed differentially at both the transcriptome and proteome levels **3**

Figure S2. KEGG and GO function analysis of 68 candidate *M. tuberculosis* gene pairs/clusters associated with drug resistance **4**

Figure S3. Identification of ETH-SO by HPLC-MS/MS **5**

Figure S4. Structure of apo-Rv3094c and Rv3094c-FMN **6**

Figure S5. Structure of Rv3094c-FMN-ETH **7**

Figure S6. Rv3094c is likely a two-component system monooxygenase **8**

Figure S7. Comparison of the active sites of FMN-binding monooxygenases **9**

Supplementary Figures

Supplementary Figure 1

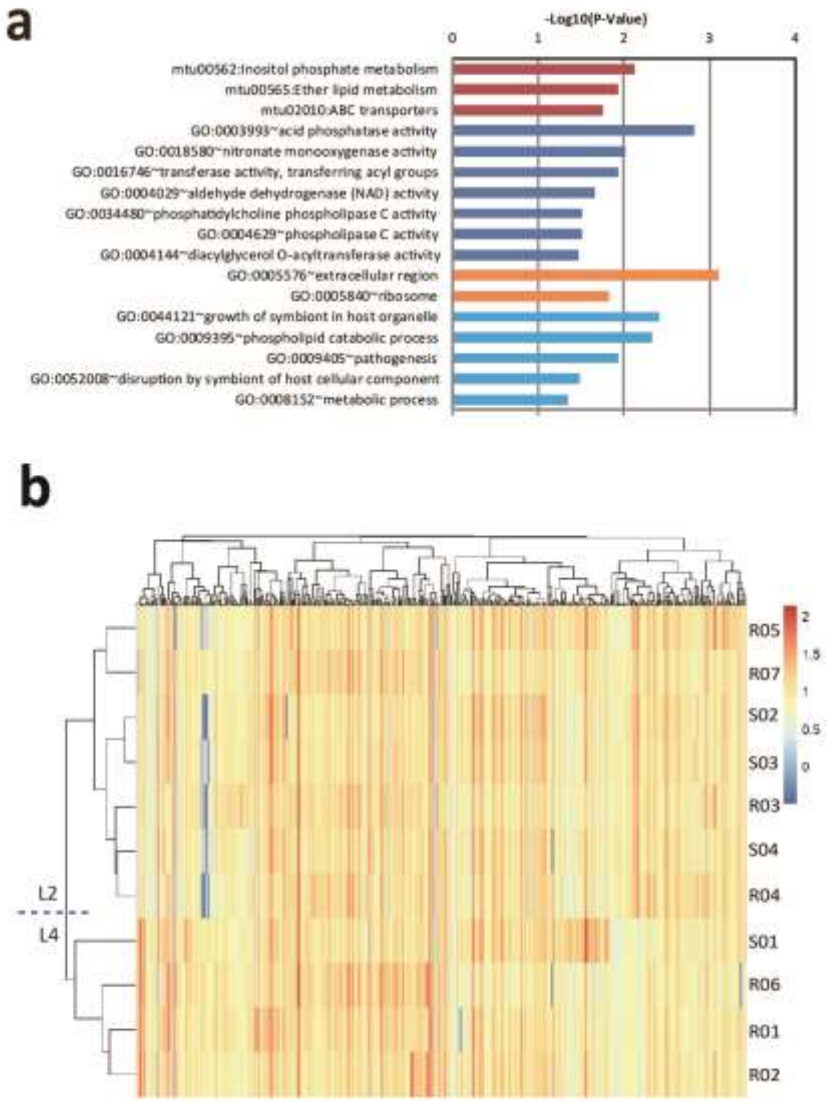

KEGG and GO function analysis of 172/388 *M. tuberculosis* genes expressed differentially at both the transcriptome and proteome levels

a KEGG and GO analysis of the 172/388 genes for which data was available. **b** Hierarchical clustering of the 11 isolates based on transcriptomic data from 373 genes differentially expressed in both the transcriptome and proteome that did not contain lineage-related SNPs.

Supplementary Figure 2

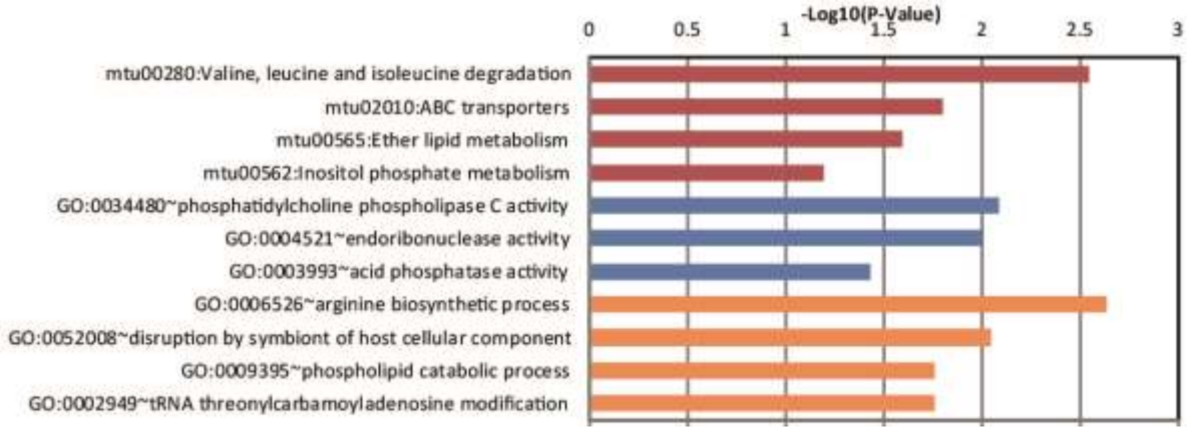

KEGG and GO function analysis of 68 candidate *M. tuberculosis* gene pairs/clusters associated with drug resistance

KEGG and GO analysis of the 109/231 genes present in the 89 gene pairs/clusters for which data was available.

Supplementary Figure 3

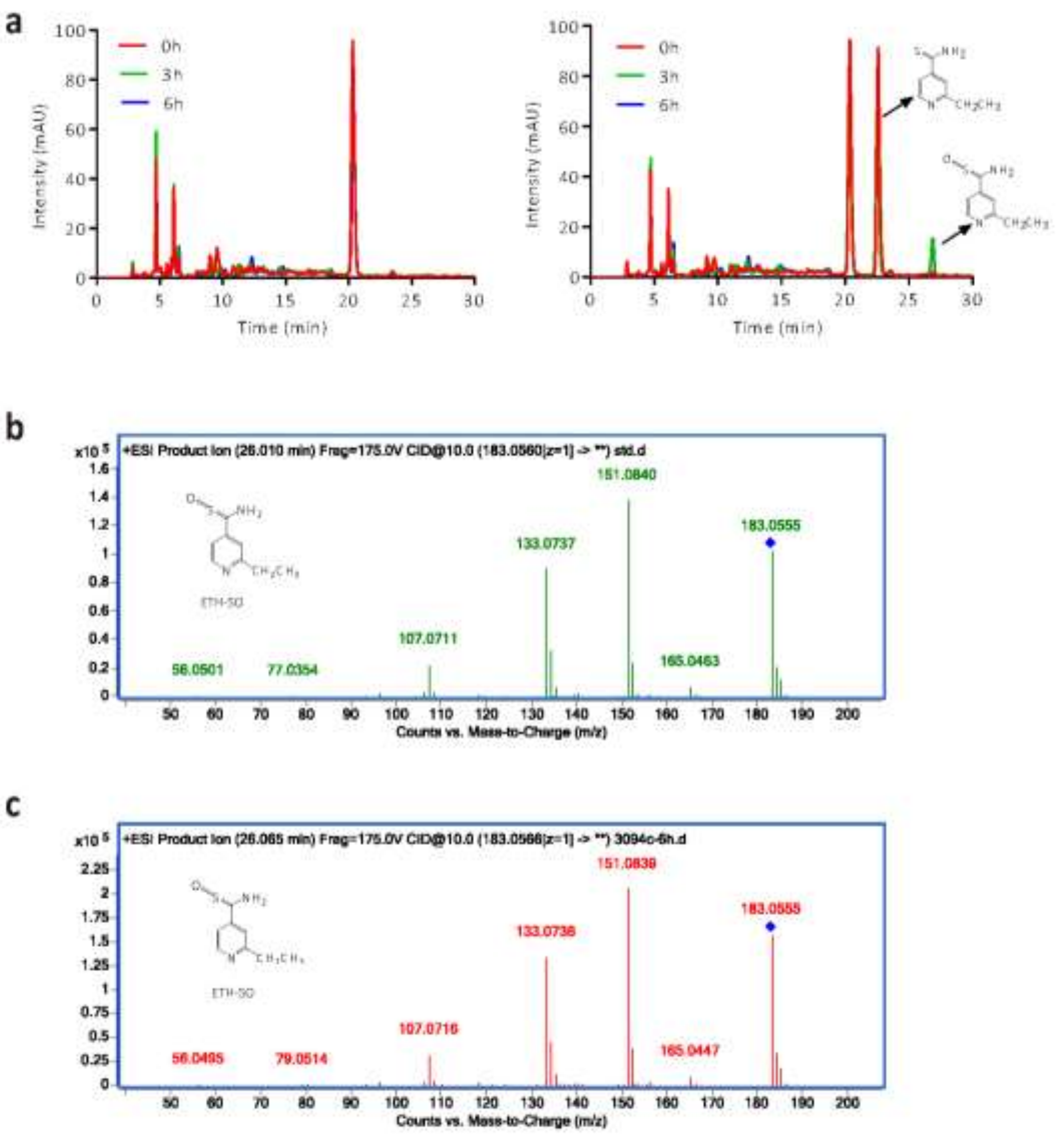

Identification of ETH-SO by HPLC-MS/MS

a HPLC spectra for *E.coli* BL21 harboring pET28a-ethA without addition of ETH (left) and incubated with 80 μg/ml of ETH (right). **b** Secondary MS profiles for an ETH-SO standard and **c** The metabolite corresponding to the peak at 27.1 min in the HPLC-UV profile of an *E. coli* strain overexpressing Rv3094c incubated with ETH for 6 h in Fig 4c.

Supplementary Figure 4

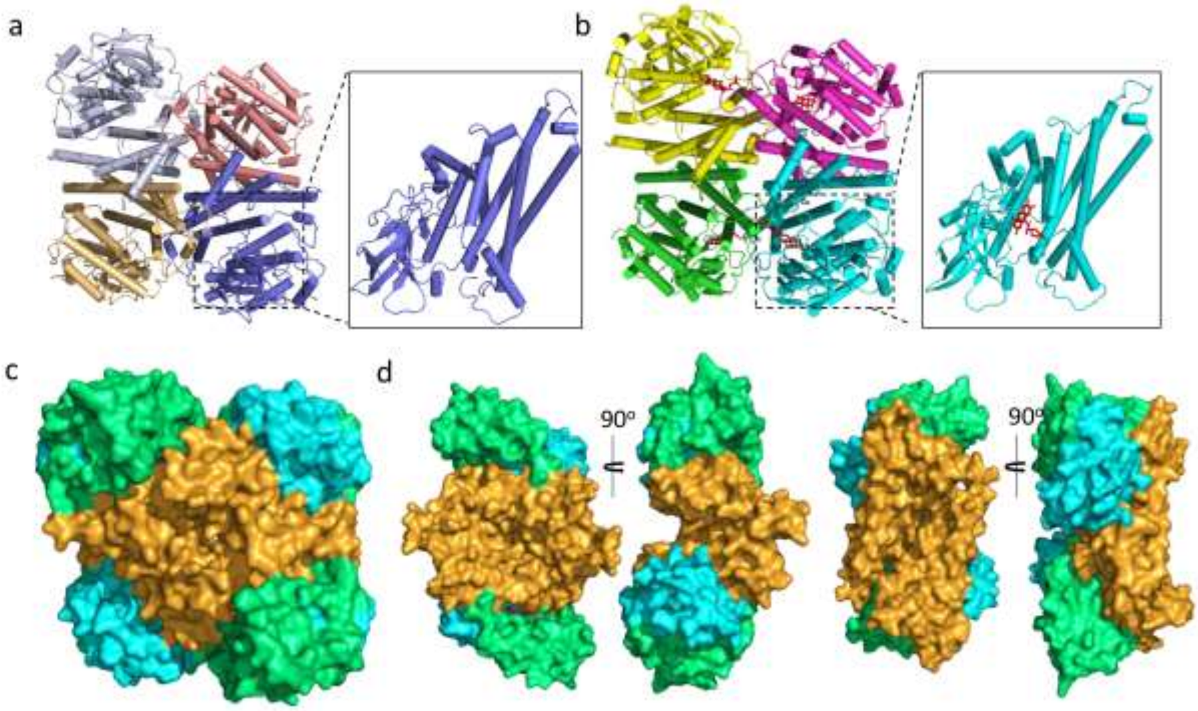

Structure of apo-Rv3094c and Rv3094c-FMN

a Ribbon diagram of the apo-Rv3094c tetramer and monomer. Four identical monomers in the tetramer are shown in gray, wheat, slate and salmon, respectively. **b** Ribbon diagram of the Rv3094c-FMN tetramer and monomer. Four identical monomers are shown in cyan, magenta, yellow and green, respectively. FMN is shown in red. **c** Surface representations of the Rv3094c-FMN-ETH tetramer. Cyan: N-terminal domain; green: middle domain; orange: C-terminal domain. **d** Surface representations of the interactions between Rv3094c monomers. Cyan: N-terminal domain; green: middle domain; orange: C-terminal domain.

Supplementary Figure 5

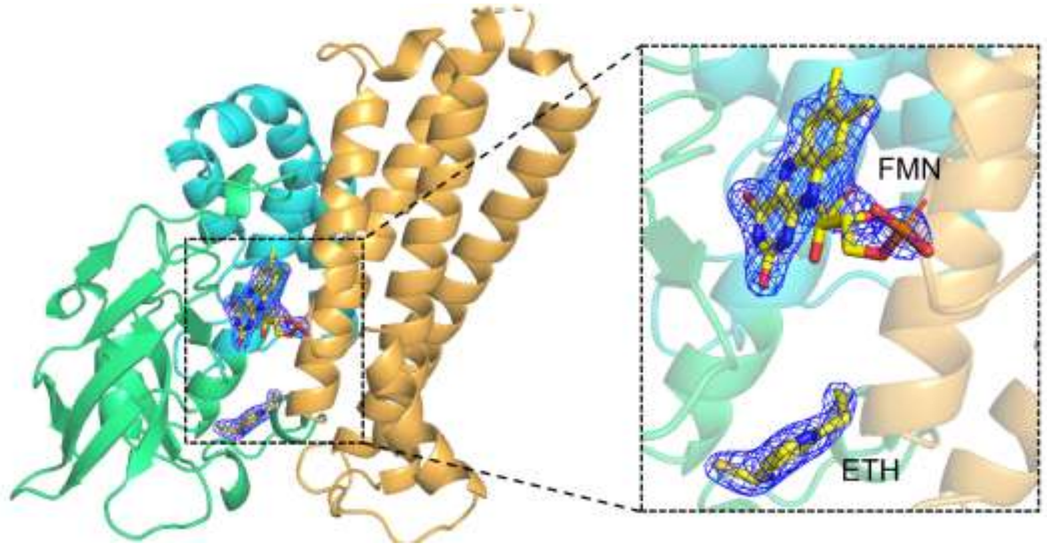

Structure of Rv3094c-FMN-ETH

Electron density when both FMN and ETH are bound to Rv3094c. FMN and ETH are represented as sticks. The 2Fo-Fc omit map is contoured at the 1.0 σ level. Carbon is shown in yellow, nitrogen in blue, and phosphorus in red.

Supplementary Figure 6

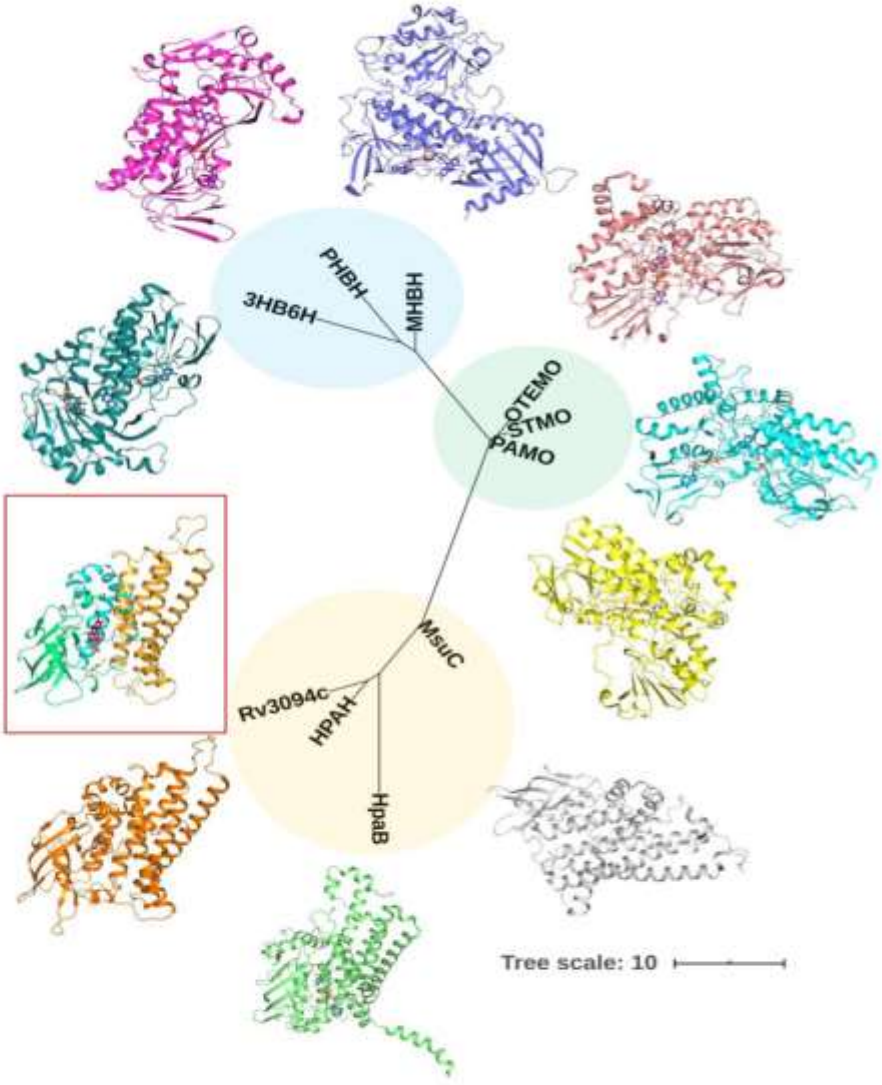

Rv3094c is likely a two-component system monooxygenase.

Structure-based phylogenetic tree of Rv3094c and related monooxygenases. The Rv3094c structure was compared with two-component monoxygenases MsuC (*Pseudomonas fluorescens*, PDB: 6UUG), HpaB (*Thermus thermophilus* HB8*i*, PDB: 2YYG) and HPAH (*Acinetobacter baumann*, PDB: 2JBS), one-component monooxygenases 3HB6H (*Rhodococcus jostii* RHA1, PDB: 5HYM), PHBH (*Pseudomonas fluorescens*, PDB: 1PBE) and MHBH (*Comamonas testosterone*, PDB: 2DKH), and Baeyer-Villiger monooxygenases OTEMO (*Pseudomonas putida*, PDB: 3UOZ), STMO (*Rhodococcus rhodochrous*, PDB: 4AOS) and PAMO (*Thermobifida fusca*, PDB: 1W4X). Rv3094c clusters with two-component monooxygenases. The structure-based phylogenetic tree was calculated using the mTM-align (https://yanglab.nankai.edu.cn/mTM-align/) and IQ-TREE (http://iqtree.cibiv.univie.ac.at/) web servers.

Supplementary Figure 7

a

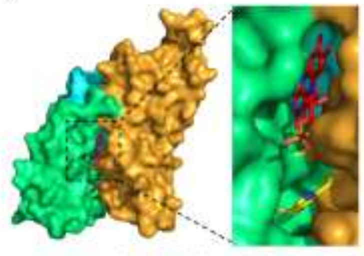

b

Protein	RMSD	Sequence identity	PDB ID
Flavin-dependent bacterial indoloterpenoid cyclase	1.9 Å	28%	5LVU
Hydroxyphenylacetate (HPA) hydroxylase	1.9 Å	23%	2JBR
Dibenzothiophene monooxygenase	2.5 Å	20%	5XDG

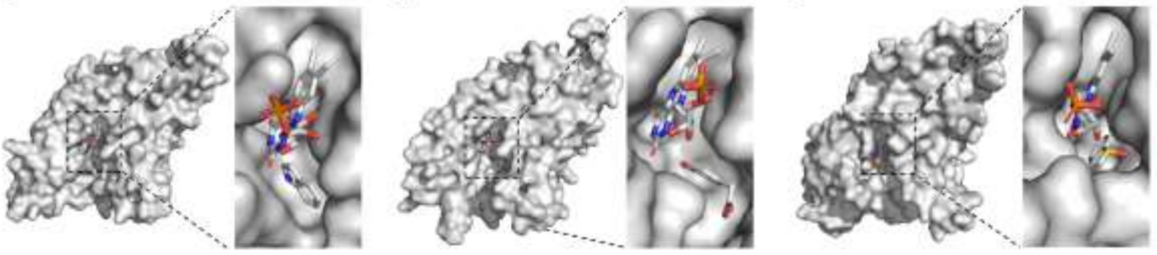

Comparison of the active sites of FMN-binding monooxygenases.

a The substrate-binding site of the Rv3094c-FMN-ETH complex. **b** Apo-Rv3094c homologs identified in a search for published structural homologs on the EMBL Dali server (http://ekhidna2.biocenter.helsinki.fi/dali/). **c** The substrate-binding site of *Streptomyces spp*. indole hydroxylase HKI0576 (PDB ID: 5lVW). **d** The substrate-binding site of *Acinetobacter baumannii* 4-HPA 3-monooxygenase large component (PDB ID: 2JBT). **e** The substrate-binding site of dibenzothiophene monooxygenase from the thermophile *Paenibacillus spp.* A11-2 (PDB ID: 5XDG).
