# Supplementary material for: Omics analysis of Mycobacterium tuberculosis isolates uncovers Rv3094c, an ethionamide metabolism-associated gene
Source: Commun Biol. 2023 Feb 7;6:156. doi: 10.1038/s42003-023-04433-w (PMC9904262; doi:10.1038/s42003-023-04433-w)
Supplement: Supplementary file 2 — Description of Additional Supplementary Files [file 42003_2023_4433_MOESM2_ESM.pdf]

Description of Additional Supplementary Files

File name: Supplementary Data 1.
Description: Background information on the 11 *Mycobacterium tuberculosis* isolates analysed in this study.

File name: Supplementary Data 2.
Description: a. SNPs present in the 11 *M. tuberculosis* isolates analysed in this study. b. 130 lineage-related SNPs. Lineage-related SNPs were present in all L2 isolates and absent in all L4 isolates. c. SNPs present in 11 well-known DR-associated genes in the 11 isolates.

File name: Supplementary Data 3.
Description: Transcriptome data for the 11 *M. tuberculosis* isolates analysed in this study. Values given are FPKM values for three biological replicates of each isolate.

File name: Supplementary Data 4.
Description: a. TMT quantitative proteomic analysis of *M. tuberculosis* isolates in Group 1. b. TMT quantitative proteomic analysis of *M. tuberculosis* isolates in Group 2.

File name: Supplementary Data 5.
Description: a. Number of times a gene was up-regulated in each L2 strain relative to each L4 strain in transcriptome comparisons. b. Number of times a gene was down-regulated in each L2 strain relative to each L4 strain in transcriptome comparisons. c. Number of times a protein was up-regulated in each L2 strain relative to each L4 strain in proteome comparisons among group 1 isolates. d. Number of times a protein was down-regulated in each L2 strain relative to each L4 strain in proteome comparisons among group 1 isolates. e. Number of times a protein was up-regulated in each L2 strain relative to each L4 strain in proteome comparisons among group 2 isolates. f. Number of times a protein was down-regulated in each L2 strain relative to each L4 strain in proteome comparisons among group 2 isolates.

File name: Supplementary Data 6.
Description: Gene IDs of the 388 *M. tuberculosis* lineage-related genes.

File name: Supplementary Data 7.
Description: KEGG and GO analysis of 172/388 genes differentially expressed at both transcriptome and proteome levels for which data was available.

File name: Supplementary Data 8.
Description: a. Gene pairs and clusters showing differential RNA expression between L2 DS and DR isolates. b. Gene pairs and clusters showing differential RNA expression between L4 DS and DR isolates.

File name: Supplementary Data 9.
Description: a. SNP genomic locations and gene and protein expression changes in DR:DS comparisons among L2 isolates. b. SNP genomic locations and gene and protein expression changes in DR:DS comparisons among L4 isolates.

File name: Supplementary Data 10.
Description: GO and KEGG analysis of the 68 gene pairs/clusters.

File name: Supplementary Data 11.
Description: Presence of predicted operons in the 68 gene pairs/clusters.

File name: Supplementary Data 12.
Description: a. Effects of the overexpression of selected candidate DR-associated gene pairs/clusters on minimal inhibitory concentrations against seven anti-tubercular drugs. b. Effects of the overexpression of selected DR-associated genes on minimal inhibitory concentrations against three anti-tubercular drugs.

File name: Supplementary Data 13.
Description: MIC analysis of an *M. tuberculosis* H37Ra strain overexpressing Rv3094c against ethionamide.

File name: Supplementary Data 14.
Description: Analysis of Rv3094c and Rv3093c expression in *M. tuberculosis* H37Ra overexpressing Rv3095. a. Relative quantification of Rv3093c expression by qPCR. b. Relative quantification of Rv3093c expression by RNA-seq. c. Relative quantification of Rv3093c expression by qPCR. d. Relative quantification of Rv3093c expression by RNA-seq.

File name: Supplementary Data 15.
Description: Ethionamide bioactivation activity of Rv3094c wild type protein and its mutants.
